# Traditional knowledge in semi-rural close to industrial areas: ethnobotanical studies in western Gironès (Catalonia, Iberian Peninsula)

**DOI:** 10.1186/s13002-019-0295-2

**Published:** 2019-04-02

**Authors:** Airy Gras, Ginesta Serrasolses, Joan Vallès, Teresa Garnatje

**Affiliations:** 1 Laboratori de Botànica (UB) – Unitat associada al CSIC, Facultat de Farmàcia i Ciències de l’Alimentació, Universitat de Barcelona, Avinguda Joan XXIII 27-31, 08028 Barcelona, Catalonia Spain; 2 Institut Botànic de Barcelona (IBB, CSIC-ICUB), Passeig del Migdia s/n, Parc de Montjuïc, 08038 Barcelona, Catalonia Spain; 3 Institut de Recerca de la Biodiversitat (IRBio), Universitat de Barcelona, Avinguda Diagonal 643, 08028 Barcelona, Catalonia Spain; 4 Secció de Ciències Biològiques, Institut d’Estudis Catalans, Carrer del Carme 47, 08001 Barcelona, Catalonia Spain

**Keywords:** Ethnobotany, Ethnoflora, Gironès, Medicinal uses, Plant uses, Traditional knowledge

## Abstract

**Background:**

The western Gironès is a district located in NE Catalonia (NE Iberian Peninsula). This area comprising 186.55 km^2^ and 10,659 inhabitants is composed of 5 municipalities encompassing 29 villages, located in the hydrographic basins of the Ter and Llémena rivers.

**Methods:**

Following the methodology based on the semi-structured interviews, we carried out 40 interviews with 57 informants, 31 were women and the remaining 26 were men, with an average age of 78.6 years.

**Results:**

In the present study, data from 316 taxa (301 angiosperms, 8 gymnosperms, and 7 pteridophytes) belonging to 89 botanical families were collected. The interviewed informants referred 3776 UR of 298 taxa, 1933 (51.19%) of them corresponding to the food category, 949 (25.13%) to the medicinal ones, and 894 (23.68%) to other uses. In addition, 581 vernacular names for 306 species, subspecies, and varieties have also been collected.

**Conclusions:**

These results reveal the validity of traditional knowledge in the studied area, which can be seriously threatened by the loss of its rural condition and its proximity to industrialized areas.

## Introduction

The Catalan-speaking territories constitute a cultural unity that has attracted the interest of researchers from various disciplines. Since two pioneering PhD theses [1, 2], several similar studies have been devoted to ethnobotanical research in these areas ([3–14], among others), and papers derived from these academic works) with the common objective of collecting, inventorying, preserving, and disseminating the popular uses of plants. The so-called acculturation process taking place in the industrialized areas, in other words the adoption of modern culture to the detriment of the traditional one [15], is the main cause of the loss of this knowledge, which must be available for future generations.

For this reason, the research that was initially focused on non-industrialized areas [16–22] has now been expanded in industrialized areas due to their rapid loss of traditional knowledge [23–27].

Although ethnobotany, as defined by Harshberger [28], was conceived to study the plants used by a particular human group—not limited to any type of use—most studies have placed special interest in medicinal plants [29–31] and secondly in those used for food purposes [32–36]. The studies comprising the whole ethnobotanical knowledge of an industrialized area are less frequent. This situation is explained, according to Gras et al. [37], due to the fact that medicinal and food uses are most related to human health, which is still valid despite the above-mentioned acculturation process. In addition, according to these authors, plants with medicinal and food uses are more susceptible to being potentially used or transformed into commercial products.

**Fig. 1:**
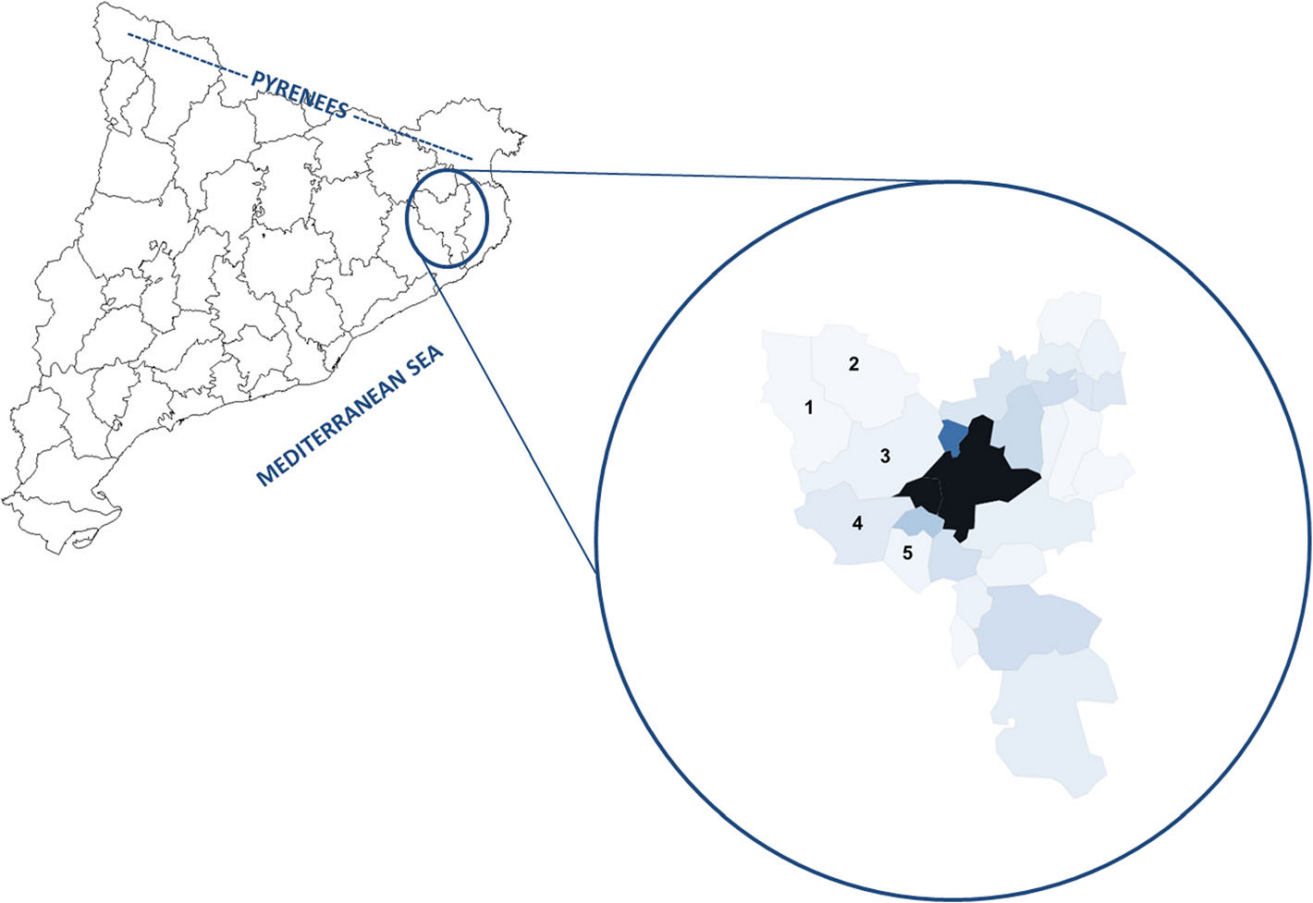
Location of the studied areas. In blue, Gironès district, including the study area (western Gironès). The figures correspond to the municipalities studied. 1: Sant Martí de Llémena, 2: Canet d’Adri, 3: Sant Gregori, 4: Bescanó, and 5: Aiguaviva. The intensity of colors is related to population density

The district (in Catalan “comarca”) of Gironès is located in NE Catalonia (Fig. 1), in its turn situated in the NE Iberian Peninsula. Our study was centered on the western part of this district, considering the natural unit constituted by territories under the influence of the hydrographic basins of the Ter and Llémena rivers. The western Gironès is composed of 5 municipalities encompassing 29 villages. The study area comprises 186.55 km^2^ and 10,659 inhabitants [38] representing a density of 57.14 inhabitants/km^2^. The altitudes range from 102 m a.s.l. in the locality of Bescanó to 256 m a.s.l. in Sant Martí de Llémena.

The Gironès district has a Mediterranean climate with an irregular rain distribution with relatively wet springs and autumns and dry summers and winters. The mean rainfall increases in SE-NW direction, with values around 1000 mm per year in the North-Western edge. Winters are moderately cold and summers are hot, with an annual mean of 14.4 °C [39].

The landscape of the area was described by Girbal [40] and is very heterogeneous, the low areas are occupied by dry lands, with herbal communities from the alliances *Diplotaxion erucoidis* and *Secalion cerealis.* In the mountainous regions, there is an altitudinal gradient, from the calcicolous scrubs of *Rosmarino*-*Ericion* with *Pinus halepensis*. in the lowlands to the beeches with Pyrenean squill (*Scillo liliohyacinthi*-*Fagetum sylvaticae*) in the highlands. The intermediate zones are occupied by holm oak forests (*Viburno tini*-*Quercetum ilicis* subass. *pistacietosum* and *Asplenio*-*Quercetum ilicis*) and by a narrow belt of oak (*Quercus pubescens*) in the upper part connecting with beech (*Fagus sylvatica*).

Economically, this area has evolved through different historical periods: prior to the industrial era, it was based on agriculture—mainly cereals—livestock, and forest management for timber and charcoal production and a second period based on textile industry. Currently, these villages do not have their own economy and they have become dormitories for people working in Girona, the capital of the district, with an important economic activity [38]. To sum up, western Gironès is still at least what can be called a semi-rural area, since agriculture is still alive there, but three of its municipalities (Aiguaviva, Bescanó, Sant Gregori) play the above-mentioned role of dormitory to the close metropolitan, industrial area. Additionally, the river Llémena valley hosts an important number of secondary residences for people from the neighboring territory, especially from Girona, the 11th biggest city in Catalonia, with a population very close to 100,000, and head of one of the four Catalonian administrative units (province) including several districts, as among which the one here considered [38].

The main goals of the present study were (i) to collect plant uses and their vernacular names in a semi-rural area, to inventory and preserve this knowledge in order for it to be available to future generations, and (ii) to analyze the obtained results in order to establish some comparisons with similar territories.

## Material and methods

### Field work

**Fig. 2:**
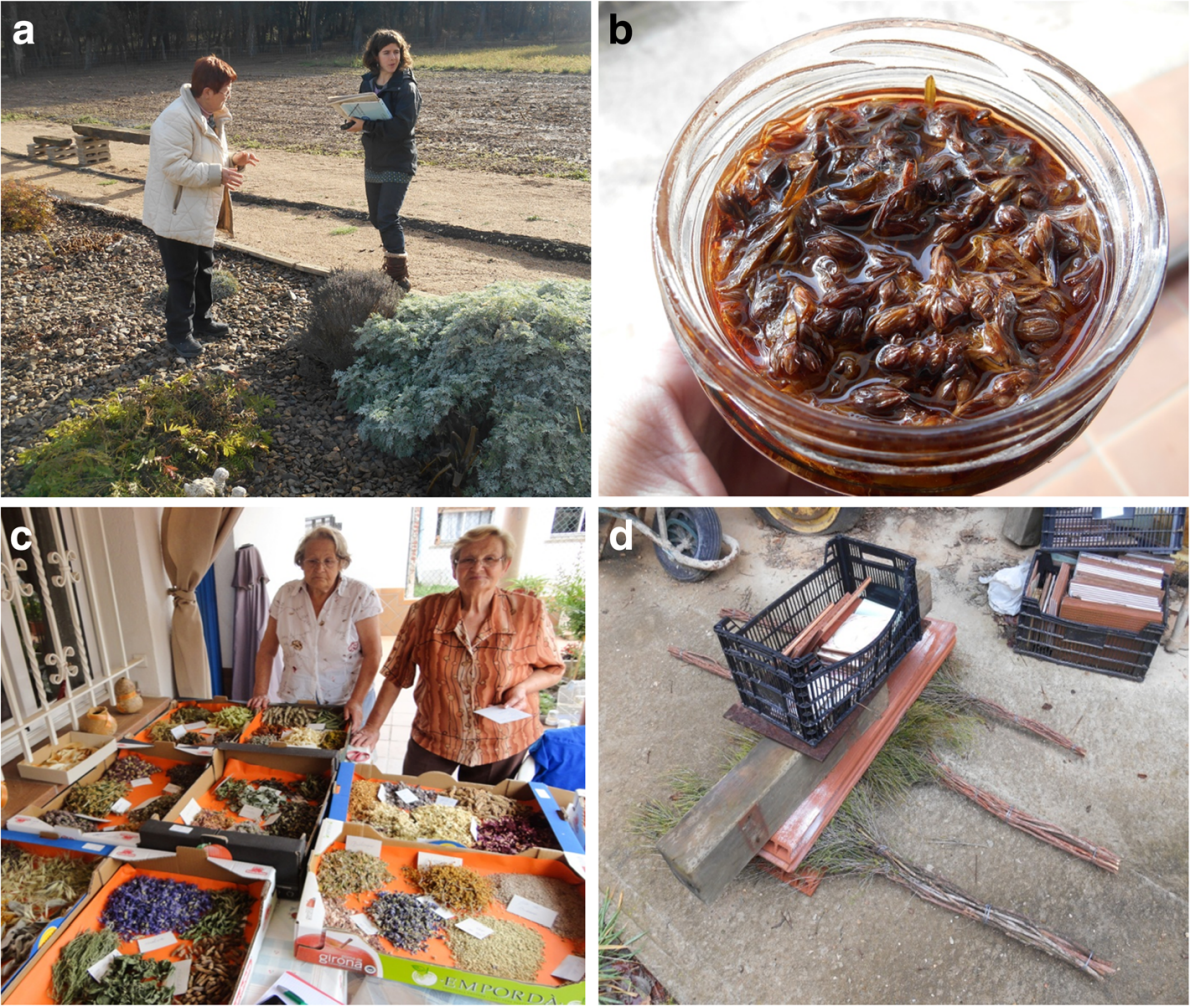
Exemples of ethnobotanical interviews and some products derived from plants. **a** Interview in a homegarden. **b** *Hypericum perforatum* oil for burns and bumps. **c** Informants with ingredients to prepare *ratafia*. **d** *Erica scoparia* brooms

The fieldwork took place from June 2013 to August 2014. We carried out 40 interviews to 57 informants: 23 were individual and 17 concerned 2 people, no one implying a bigger group. Out of the interviewed people, 31 (54%) were women and the remaining 26 (46%) were men. The methodology used was based on the semi-structured interviews [41] avoiding closed questionnaires and direct questions that could have an implicit answer so as not to coerce informants’ answers (Fig. 2). Conversations were developed in the Catalan language, common to interviewers and interviewees. During the ethnobotanical surveys, we not only focused on medicinal and food uses but also asked for knowledge of plants with other uses. The popular names of plants, in Catalan, were also collected.

We have recorded information on both wild and cultivated plants, and also on plants that can be bought through commerce. Results are presented according to the classification of the folk uses of the species in three main categories: medicinal, food, and other uses. Within the food category, we distinguish the human and animal uses. To define the types of medicinal plant uses, we basically follow Cook’s Economic Botany Data Collection Standard [42].

The plant taxa cited by the informants were identified using the *Flora dels Països Catalans* [43] and the *Flora Manual dels Països Catalans* [44], which we basically follow for nomenclature. The allocation of families has been done following the APG IV [45]. The herbarium vouchers have been deposited in the herbarium BCN (Centre de Documentació de Biodiversitat Vegetal, Universitat de Barcelona).

The field work respected the ethical principles of the International Society of Ethnobiology [46] and we had the prior oral informed consent of the informants [47].

### Data analysis

The interviews were recorded and subsequently transcribed, and all the information obtained was entered into the database of our research group (www.etnobotanica.cat). The analyses were carried out with Excel (Microsoft Excel 2007) and XLSTAT (v2007.5, Addinsoft SARL) programs. To analyze the results, we have used the use report (hereinafter, UR) [48].

With the aim of assessing the state of knowledge, studies of quantitative ethnobotany were also performed and the following indices were calculated: ethnobotanicity index (EI; [49]), which is the quotient between the number of plants used and the total number of plants that constitute the flora of the territory, expressed as a percentage; the informant consensus factor (F_IC_; [50]), which is the quotient between the number of medicinal use reports minus the number of used medicinal plants and the number of medicinal use reports minus one. This indicates the degree of reliability of the uses claimed (higher when closer to 1).

Number of medicinal plants used per informant (P/I), per inhabitant (P/H), and per unit of area (P/km^2^) were calculated, in order to compare with other territories from which this information is provided only for this kind of useful plants. The linguistic diversity index [51], obtained by dividing the number of folk names by the number of taxa reported, has been calculated to illustrate the cultural richness of the folk plant knowledge.

Finally, we calculated the recently proposed index of taxon usefulness in mixtures (ITUM; [52]), which is the quotient between the number of citation of this taxon in mixtures and its total citations, whether with simple or complex presentation. This index indicates the exclusiveness of taxa in mixtures when the value is one or closer to one.

## Results and discussion

This study contributes information to complete the ethnobotanical knowledge in the North Eastern Catalan linguistic and cultural area, where still a territory is to be investigated before being able to perform a meta-analytic work. It also enlarges the ethnofloristic knowledge of the Iberian territories, which are among the most studied in Europe [27]. We believe that, in general, increasing data on Catalan and Iberian folk plant knowledge provides them with a bigger robustness, apart from contributing new or rare uses and taxa used. Plants having appeared not very long time ago in European folk phytotherapy constitute not the only but a good example of such additions that prospects as the present one can bring to the ethnoflora. Although when first contacting the informants we indicate that we are interested in orally-transmitted traditional uses, in some cases, they report to us that a certain knowledge on a plant use is recently acquired. Just as a case example, *Aloe vera* does not appear in the pioneering works on Catalan ethnobotany [1, 2], but is importantly present, with ten use reports, in this one. Even if these data may have not been considered in some occasions, a reflection should be initiated on the new incorporations to folk knowledge, which will become tradition and will lead to a renewed paradigm in plant uses.

### Characteristics of the interviewees

The average age of the informants is 78.6 years, ranging from 58 to 92, the interval between 78 and 80 years being the one that accumulates a greater number of informants. This average is one of the highest values found in the recent studies carried out in similar areas and only surpassed in the island of Formentera [11].

Most informants were native (74%) and the remaining ones have lived in the area for more than half of their lives. Only 10% are native from the neighboring district of la Selva.

Regarding their work, most of the men have been farmers (18%) or shepherds (5%), while most of the women have combined the farm work with household affairs (23%). Other professions linked to the territory are textile (16%) and hotel (7%) industries, both important economic activities in this area.

### Plant species, use reports, and botanical families

Data from 316 taxa (301 angiosperms, 8 gymnosperms, and 7 pteridophytes) belonging to 89 botanical families were collected in the present study. Thirteen taxa have only been determined at generic level and 19 present infraspecific categories. In the first case, taxa—in fact ethnotaxa—were referred to by the informants without specific category. It could be due to several or all species of the genus being used, or to the fact that they were not able to distinguish the taxa. The complete catalog of the recorded useful plants in the studied area is contained in Serrasolses [13], and the data concerning all plants, shown later, are synthesized, arranged by large use categories.

The five best represented families are Lamiaceae (12.39%), Poaceae (9.25%), Rosaceae (7.35%), Asteraceae (6.84%), and Fabaceae (5.55%), which partially coincides with the findings in other territories with similar characteristics [6, 10, 12, 53] and at the same time represents the most common botanical families, apart from Apiaceae and Rutaceae, of the Mediterranean flora [44]. This fact links with the idea that the closer to civilization a plant grows, the more it is used by local people [54–57].

The interviewed informants refer 3776 UR of 298 taxa, 1933 (51.19%) of them corresponding to the food category, 949 (25.13%) to the medicinal ones, and 894 (23.68%) to other uses. The mean of UR per informant is 66.25, and 5.23 taxa per informant are cited, but these values show very large deviations due to the differences in knowledge that exist between the informants.

Medicinal plants are the most reported in the majority of ethnobotanical works carried out in the Catalan Countries [6, 10, 58]. However, in the present study, food uses are the most cited by the informants due to the collection of a large number of recipes devoted to the preparation of *ratafia* (see comments on this beverage in 3.5). This traditional Catalan liqueur [59], prepared with the immature fruit of *Juglans regia* and numerous species of preferably aromatic plants, is still consumed in areas close to the study area [60].

### Quantitative ethnobotany

Some quantitative ethnobotany indexes concerning ten territories (the one here studied included) of the Catalan linguistic area are presented in Table 1. The ethnobotanicity index, not having into account the 50 taxa of allochthonous plants recorded, is 22.56% for the studied area; this roughly meaning that between one-fifth and one-quarter of the plants of the area have been claimed as useful by the informants. It occupies an intermediate position in the range of the values obtained for other Catalan-language studied areas The informant consensus factor (F_IC_) of medicinal information obtained for our interviewees (0.86) is close to the highest values in the quoted areas. Interestingly, this value, accounting for the consistency (thus, reliability) of plant use within a cultural and geographical group, which is an indicative of a generationally transmitted knowledge is higher to those obtained in Mexican areas (0.75, 0.79; [61, 62]). Recently, an ethnobotanical study of medicinal foods used by practitioners in an Indian area shows F_IC_ for the different ailments treated ranging from 0 to 1, but low in mean value (0.26; [63]). The results are similar (with a highest value of 0.72) in a study of medicinal plants in the Greek Aegean Islands [64]. This indicates that the traditional pool of knowledge on plant use and management is still alive in the studied area. Consequently, we can state that there is a high consistency in folk plant knowledge in the industrial European zone considered, where it could have been hypothesized it would be lower, even as compared with less industrialized Asian or American territories, where ethnobiological data are a priori supposed to be high, robust, and less eroded.

**Table 1:** Quantitative ethnobotany indexes in ten territories (in italic, the one here studied) in the Catalan linguistic area. EI: ethnobotanicity index; F_IC_: informant consensus factor; MP: number of medicinal plants

Territory	EI	F_IC_	MP/informant	MP/inhabitant	MP/km^2^
Alt Empordà [10]	25.90	0.91	1.88	0.28 × 10^−2^	0.25
Castelló [1]	15.00	–	2.34	0.06 × 10^−2^	0.06
Cerdanya [2, 7]	–	0.93	1.11	0.82 × 10^−2^	0.23
Segarra [76]	–	–	3.17	0.54 × 10^−2^	0.13
Eastern Mallorca [57]	15.51	0.71	2.88	0.38 × 10^−2^	0.51
*Western Gironès* (this paper)	*22*.*56*	*0*.*86*	*2*.*40*	*1*.*29* × *10*^−2^	*0*.*73*
Guilleries [4]	20.00	–	5.64	0.58 × 10^−2^	0.27
Montseny [6]	23.20	0.91	1.95	0.44 × 10^−2^	0.42
Pallars Jussà and Pallars Sobirà [5]	29.10	0.87	1.66	2.32 × 10^−2^	0.16
Ripollès [58]	28.60	0.96	1.73	1.10 × 10^−2^	0.29

### Medicinal uses

Our informants mentioned 137 species with medicinal uses and 949 use reports, 81.66% of which are referring to human medicine, 7.06% to veterinary, and 1.37% to both human and veterinary medicines (Table 2). No information was reported for the remaining 9.91%. The mean of medicinal taxa cited by informant is 2.40. This number of medicinal plants, quoted by the 57 informants, is close (slightly lower in ratio taxa/informant) to the one found in an area covering a part of the island of Mallorca, with a comparable number of interviewees as well: 121 taxa quoted by 42 informants [57]. Conversely, a recent study in a Turkish region [65] reports 92 taxa (35% of which with medicinal uses) quoted by 123 informants, i.e., a clearly lower ratio. Similarly, a research in a Myanmar area [66] records 75 medicinal taxa cited by 206 informants. This is also the case in Europe: in the Greek Aegean Islands, 200 informants reported uses of 109 medicinal plants [64]; the authors state that these plants are used, but they do not mention any other plant quoted by the informants and not currently used. This reinforces the above-exposed argument that the ethnobotanical corpus is still relevant in industrialized areas, even in comparison with non- or less-industrialized territories, where the weight of this knowledge is a priori supposed to be higher. There is still time left (probably in its very end) to collect the traditional knowledge on plant uses in industrialized zones, which is basic in order to reintroduce it to the younger generations, or to use certain information to develop a new useful product of higher reach.

**Table 2:** Medicinal plants reported in the studied area

Family	Taxon (voucher)	Catalan vernacular names	Medicinal use	Part used	Pharmaceutical form	UR
Adoxaceae	*Sambucus nigra* L. (BCN113595)	Sabuquer. Saüc. Saüquer	Anticatarrhal. antidiarrhoeal. anti-inflammatory. antipneumonic. antipyretic. buccal antiseptic. external antiseptic. for amygdalitis. for earache. for headache. emmenagogue. expectorant. not reported. ocular antiseptic, refrigerant. stomachic	Fruit. inflorescence. not reported	Aerosol. bath. essence. eyedrops. fumigation. medicinal wine. not reported. poultice. syrup. tisane	81
Amaranthaceae	*Beta vulgaris* L. subsp. *vulgaris* var. *crassa* (Alef.) Helm (BCN50761)	Bleda. Polpa (elaborated product). Remolatxa. Sucre (elaborated product). Sucre candi (elaborated product)	Against taeniasis. anticatarrhal. antihelminthic. expectorant	Root	Decoction. direct use. ointment	8
Amaryllidaceae	*Allium cepa* L. (BCN28655)	Ceba	Antitussive. expectorant. for aphonia. not reported. resolutive	Bulb	Direct use. gargle. poultice	7
	*Allium sativum* L. (BCN29832)	All	Analgesic. callicide. for earache. hematocathartic	Bulb	Alcoholic tincture. direct use. embrocation	29
Anacardiaceae	*Pistacia lentiscus* L. (BCN29907)	Llentiscle	Analgesic. teeth strengthening	Aerial part. not reported	Collutorium	2
Apiaceae	*Conium maculatum* L. (BCN32171)	Cicuta	Anticolitic	Aerial part	Bath	1
	*Eryngium campestre* L. (BCN31274)	Espinacal	Anticholesterolemic. buccal antiseptic	Aerial part. root	Collutorium. tisane	5
	*Foeniculum vulgare* Mill. (BCN26350)	Fonoll	Anticolitic. antidiarrhoeal. digestive. galactogene^a^. internal antiseptic^a^, laxative. postpartum coadjuvant^a^. stomachic^b^	Aerial part	Direct use. emulsion. tisane	15
	*Petroselinum crispum* (Mill.) Hill (BCN29905)	Julivert	Abortive. hypoglycaemic	Aerial part. stem	Direct use	2
Araceae	*Arum italicum* Mill. (BCN32358)	Xàrria. Xèrria	Against tinea^a^. antihaemorrhoidal. anti-inflammatory. antipyrotic^b^. for amygdalitis. for skin disorders^a^	Bulb. fruit	Embrocation. not reported. ointment	6
Araliaceae	*Hedera helix* L. (BCN29869)	Heura. Heura d’alzina	Antihypertensive. antipyrotic	Leaf	Poultice. tisane	3
Asparagaceae	*Agave americana* L. (BCN46860)	Figuerassa	Not reported	Leaf	Not reported	1
	*Ruscus aculeatus* L. (BCN29939)	Galzeran. Galleranc	Cardiotonic	Root	Not reported	1
Asphodelaceae	*Aloe maculata* All. (BCN50760)	–	Antipyrotic	Leaf	Direct use	1
	*Aloe vera* (L.) Burm.f. (BCN27242)	Àloe. Àloe vera	Antipyrotic. laxative. vulnerary	Leaf. inflorescence	Direct use. embrocation	10
Aspleniaceae	*Ceterach officinarum* DC. in Lam. et DC. (BCN29850)	Dauradella	Antihypertensive. blood pressure regulator	Frond	Not reported. tisane	2
Asteraceae	*Achillea ageratum* L. (BCN113701)	Herba del fàstic	Purgative	Inflorescence	Tisane	1
	*Achillea millefolium* L. (BCN113708)	Cordonet. Herba de les milfulles	Antineoplastic. emmenagogue	Inflorescence	Tisane	2
	*Arnica montana* L. subsp. *montana* (BCN29628)	Àrnica	Anti-ecchymotic. antalgic/anti-ecchymotic/anti-inflammatory. external antiseptic. for stings	Inflorescence	Embrocation. lotion. not reported	13
	*Artemisia absinthium* L. (BCN29837)	Artemisa. Donzell	Abortive. antihelminthic. for alcohol dishabituation	Aerial part	Alcoholic tincture. not reported	3
	*Calendula arvensis* L. (BCN29637)	Lligamans	Ocular antiseptic	Aerial part	Bath	1
	*Calendula officinalis* L. (BCN29977)	Calèndula	Anti-ecchymotic. hepatoprotective	Inflorescence	Liniment. tisane	2
	*Centaurea aspera* L. (BCN113579)	Caps de burro. Flor del sucre. Travalera	Hypoglycaemic	Aerial part	Not reported. tisane	3
	*Inula helvetica* Weber (BCN24668)	Àrnica borda	Anti-ecchymotic	Inflorescence	Lotion	1
	*Matricaria recutita* L. (BCN113594)	Camamilla. Camamilla romana	Analgesic. anticatarrhal. antihelminthic. anti-nauseating. digestive. external antiseptic. internal antiseptic. ocular antiseptic. stomachic^b^	Aerial part. inflorescence. not reported	Bath. emulsion. tisane	46
	*Santolina chamaecyparissus* L. (BCN113709)	Espernallac. Santolina	Digestive. not reported	Inflorescence. not reported	Tisane. not reported	2
	*Sonchus oleraceus* L. (BCN113723)	Lletissó. Llipsó. Llistó	Diuretic	Aerial part	Not reported	1
	*Tanacetum vulgare* L. (BCN113712)	Camamilla de muntanya	Purgative^a^	Aerial part	Direct use	1
	*Taraxacum officinale* Weber in Wiggers (BCN25948)	Dent de lleó. Xicoia	Hepatoprotective	Leaf	Direct use	1
	*Tussilago farfara* L. (BCN29964)	Pota de cavall	Antipyrotic. for undetermined illnesses	Leaf	Embrocation. not reported	2
Boraginaceae	*Lithospermum officinale* L. (BCN113576)	Herba pedrera	Hepatic lithotriptic	Aerial part	Tisane	1
	*Symphytum tuberosum* L. (BCN22606)	Consolta	Vulnerary	Bulb	Ointment	1
Brassicaceae	*Brassica napus* L. (BCN46856)	Nap. Nap de bou. Nap del camp	Restorative^a^	Root	Direct use	1
	*Brassica oleracea* L. subsp. *oleracea* (BCN32181)	Bròquil. Col. Col aloma	Analgesic	Leaf	Direct use	2
Buxaceae	*Buxus sempervirens* L. (BCN29843)	Boix	For skin disorders^a^	Aerial part	Bath	1
Cannabaceae	*Celtis australis* L. (BCN29845)	Lledó (fruit). Lledoner	Anticholesterolemic. antihypertensive. blood pressure regulator. cardiotonic	Fruit. leaf	Not reported. tisane	7
Caprifoliaceae	*Lonicera implexa* Ait. (BCN113802)	Lligabosc. Mareselva. Xuclamel	External antiseptic	Flower	Bath	1
	*Scabiosa atropurpurea* L. (BCN29947)	Escabiosa	Anti-acne. antitussive. buccal antiseptic. for scarlet fever. for measles	Aerial part. flower. not reported	Collutorium. tisane	5
	*Valeriana officinalis* L. (BCN29816)	Valeriana	Abortive. sedative	Root	Tisane	2
Caryophyllaceae	*Herniaria glabra* L. (BCN113577)	Herba de les mil granes. Mil granes	Diuretic, renal anti-inflammatory	Aerial part	Tisane	4
Cistaceae	*Cistus monspeliensis* L. (BCN36740)	Estepa. Mòdega	Antidiarrhoeal	Leaf	Tisane	1
Clusiaceae	*Hypericum perforatum* L. (BCN113597)	Flor de Sant Joan. Herba de cop. Herba de Sant Joan	Anti-ecchymotic. antipyrotic. gastric anti-inflammatory. renal anti-inflammatory. vulnerary	Aerial part. flower. not reported	Embrocation. liniment. lotion. not reported	32
Cneoraceae	*Cneorum tricoccon* L. (BCN51285)	Olivereta	Antihypertensive	Leaf	Tisane	1
Crassulaceae	*Sedum sediforme* (Jacq.) Pau (BCN29792)	–	Cicatrizing	Leaf	Direct use	1
	*Sedum telephium* L. (BCN24995)	Bàlsam	Antipyrotic	Leaf	Direct use	1
Cucurbitaceae	*Cucumis sativus* L. (BCN46850)	Cogombre (fruit)	Antihaemorrhoidal. antivaricose. gastric anti-inflammatory	Fruit	Liniment	3
	*Cucurbita pepo* L. var. *pepo* (BCN49858)	Carbassa (fruit). Carbassera. Rabequet (fruit)	Antihelminthic. for abscesses. for skin disorders. prostate anti-inflammatory. renal lithotriptic	Fruit. seed	Not reported. ointment	6
Cupressaceae	*Juniperus communis* L. (BCN113589)	Ginebre. Ginebró	Analgesic. for scabies^a^	Fructification. root	Liniment. lotion. poultice	3
	*Juniperus oxycedrus* L. (BCN29879)	Càdec	Not reported	Aerial part	Not reported	1
Equisetaceae	*Equisetum arvense* L. (BCN24767)	Cua de cavall. Sangnua	Diuretic. not reported	Aerial part	Direct use. not reported	2
	*Equisetum* sp.	Cua de cavall. Sangnua	Analgesic. antihypertensive. buccal antiseptic. diuretic. urinary antiseptic	Arial part	Collutorium. tisane	12
	*Equisetum telmateia* Ehrh. (BCN113581)	Cua de cavall. Sangnua	Diuretic. for iron-deficiency. renal lithotriptic. salutiferous	Aerial part	Tisane	8
Euphorbiaceae	*Euphorbia* sp.	Lletdetereses. Lletdetresa	For warts	Latex	Direct use	4
	*Mercurialis annua* L. (BCN29896)	Blet. Murcarol	Laxative	Aerial part	Tisane	1
	*Ricinus communis* L. (BCN46089)	Oli de ricí (elaborated product)	Purgative	Fruit	Direct use	1
Fabaceae	*Ceratonia siliqua* L. (BCN32177)	Garrofa (fruit)	Salutiferous^a^	Fruit	Direct use	1
	*Medicago sativa* L. (BCN29891)	Userda	Analgesic. anti-ecchymotic. not reported	Aerial part	Poultice	4
	*Spartium junceum* L. (BCN29956)	Ginesta	Anti-ecchymotic. insects repellent^a^	Flower	Liniment	2
Fagaceae	*Quercus ilex* L. (BCN113730)	Aglà (fruit). Alzina. Aulina. Gla (fruit)	Antibronchitic. antidiarrhoeal^a^. cicatrizing^b^. for amygdalitis	Bark. in situ living plant. leaf. stem	Bath. colloidal solution. direct use	5
Gesneriaceae	*Ramonda myconi* (L.) Reichenb. (BCN46088)	Orella d’os	Anticatarrhal. antihaemorrhoidal. antiherpes. antipneumonic. antipyretic, antitussive. pharyngeal anti-inflammatory. postpartum coadjuvant^a^. stomachic	Aerial part. leaf	Embrocation. not reported. tisane	15
Juglandaceae	*Juglans regia* L. (BCN29877)	Noguer. Nou (fruit). Nou verda (fruit)	Antialopecia. antihypertensive	Leaf	Bath. tisane	2
Lamiaceae	*Hyssopus officinalis* L. (BCN29709)	Hisop	Anticatarrhal	Aerial part	Tisane	1
	*Lavandula dentata* L. (BCN29715)	Lavanda	Anti-inflammatory	Flower	Direct use	1
	*Lavandula stoechas* L. (BCN113714)	Cap d’ase. Tomanyí	Stomachic	Flower	Tisane	2
	*Melissa officinalis* L. (BCN113713)	Melissa. Tarongina	Tranquilizer	Aerial part	Tisane	1
	*Mentha* ×*piperita* L. (BCN113813)	Menta. Menta de la xocolata. Menta piperita. Menta romana	Stomachic	Aerial part	Tisane	1
	*Mentha pulegium* L. (BCN113598)	Poliol. Poniol	Antidiarrhoeal. antihypertensive. digestive. intestinal anti-inflammatory. tranquilizer	Aerial part. flower	Tisane	24
	*Mentha spicata* L. (BCN113812)	Menta. Menta de la sopa. Menta silvestre. Menta espicata. Menta verdadera	Emmenagogue. for stings. intestinal anti-inflammatory	Aerial part. leaf	Direct use. emulsion. poultice. tisane	8
	*Origanum majorana* L. (BCN113585)	Marduix	For earache	Aerial part	Embrocation. not reported	2
	*Origanum vulgare* L. (BCN113705)	Orenga	Restorative. stomachic	Aerial part	Tisane	3
	*Prunella vulgaris* L. (BCN113578)	Herba del traïdor	Anti-acne	Aerial part	Tisane	1
	*Rosmarinus officinalis* L. (BCN113599)	Romaní	Analgesic. anticatarrhal. antidepressant. anti-ecchymotic	Aerial part	Liniment. lotion. medicinal wine. tisane	14
	*Salvia officinalis* L. subsp. *officinalis* (BCN113583)	Sàlvia. Sàlvia de fulla ampla	Analgesic. antihypertensive. for fatigue. hematocathartic. not reported. sedative	Aerial part. not reported	Collutorium. not reported. tisane	10
	*Salvia verbenaca* L. (BCN113580)	Herba de les iaies	Antipyertensive	Flower	Tisane	1
	*Satureja calamintha* (L.) Scheele (BCN113737)	Menta blava	Digestive	Aerial part	Tisane	1
	*Satureja montana* L. (BCN113741)	Sajolida	Hematocathartic	Aerial part	Tisane	1
	*Sideritis hirsuta* L. (BCN113582)	Herba de Sant Antoni	Vasotonic	Aerial part	Tisane	1
	*Stachys byzantina* C. Koch (BCN113707)	Fulles de la mare de Déu. Planta de vellut	Antipyrotic. cicatrizing. vulnerary	Leaf	Direct use. embrocation	8
	*Stachys officinalis* (L.) Trevisan (BCN25011)	Brotònica	Antihypertensive	Aerial part	Tisane	2
	*Teucrium chamaedrys* L. (BCN29806)	Brotònica	Anticatarrhal	Aerial part	Not reported	1
	*Thymus serpyllum* L. (BCN113719)	Farigola de pastor. Farigoleta. Salsa de pastor	Internal antiseptic. not reported. stomachic	Aerial part	Tisane	3
	*Thymus vulgaris* L. (BCN113590)	Farigola	Anticatarrhal. anti-inflammatory^a^. buccal antiseptic. external antiseptic^b^. gingival antiseptic. internal antiseptic^b^. not reported. ocular antiseptic. postpartum antiseptic^a^. postpartum coadjuvant^a^. salutiferous. sedative. stomachic. vulnerary^b^	Aerial part	Bath. collutorium. direct use, emulsion. fumigation. gargle. liniment. tisane	78
Lauraceae	*Cinnamomum zeylanicum* Nees (BCN47283)	Canyella	Anticholesterolemic	Bark	Direct use	1
	*Laurus nobilis* L. (BCN113717)	Llorer. Llort	Analgesic. anticatarrhal. expectorant. not reported	Leaf	Aerosol. bath. not reported	6
Liliaceae	*Lilium candidum* L. (BCN46841)	Lliri de Sant Antoni. Lliri de Sant Josep	Antipyrotic. external antiseptic. vulnerary	Flower. leaf	Embrocation. not reported	8
Linaceae	*Linum usitatissimum* L. (BCN47281)	Farina de llinet (elaborated product). Llinet	Antidiarrhoeal. buccal antiseptic. cicatrizing^a^. for abscesses. for amygdalitis. for respiratory disorders. for skin disorders. gastric anti-inflammatory. laxative. not reported. resolutive	Seed	Decoction. poultice	15
Lythraceae	*Punica granatum* L. (BCN29764)	Magrana (fruit). Magraner. Magraner agre. Magraner bord. Magraner dolç	Antihelminthic	Fruit. root	Decoction. direct use	3
Malvaceae	*Althaea officinalis* L. (BCN113799)	Malví	Not reported	Root	Not reported	1
	*Malva sylvestris* L. (BCN29889)	Malva. Malva rosa	Anticatarrhal. antipyrotic. not reported	Aerial part. flower. leaf	Not reported. poultice. tisane	3
	*Theobroma cacao* L. (BCN30763)	Xocolata (elaborated product)	Antihelminthic	Seed	Direct use	1
	*Tilia cordata* Mill. (BCN26784)	Til·la	For headache. not reported. tranquilizer	Bract with inflorescence	Tisane	5
	*Tilia platyphyllos* Scop. (BCN113739)	Tei. Til·la. Til·ler de bosc	Anticatarrhal. antihypertensive. tranquilizer	Bract with inflorescence	Tisane	8
Moraceae	*Ficus carica* L. (BCN24887)	Figa (infructescence). Figa d’Alacant (infructescence). Figa de coll de senyora (infructescence). Figa de coll llarg blanca (infructescence). Figa de coll llarg negra (infructescence). Figa de pota de cavall (infructescence). Figa de Sant Joan (infructescence). Figa negra (infructescence). Figuera. Figuera de coll de senyora	For warts	Latex	Direct use	5
Myrtaceae	*Eucalyptus globulus* Labill. (BCN29696)	Eucaliptu. Eucaliptus	Anticatarrhal. expectorant. for respiratory disorders	Leaf	Aerosol. tisane	18
Oleaceae	*Olea europaea* L. subsp. *europaea* (BCN29898)	Oli (elaborated product). Oli d’oliva (elaborated product). Olivera. Oliva (fruit)	Antihelminthic. antihypertensive. antihypotensive. antipyrotic. blood pressure regulator. cicatrizing. external antiseptic. for earache. for mastitis. for skin disorders. vulnerary	Flower. fruit. leaf	Direct use. embrocation. emulsion. fumigation. not reported. ointment. tisane	41
Paeoniaceae	*Paeonia officinalis* L. (BCN29320)	Peònia	Not reported	Root	Not reported	1
Papaveraceae	*Chelidonium majus* L. (BCN113742)	Berruguera. Celoni. Herba de les orenetes. Llet de Santa Teresa	For warts	Latex	Direct use	4
	*Papaver rhoeas* L. (BCN29903)	Gallaret. Pipiripip. Quiquiriquí. Rosella	Analgesic. sedative	Seed	Direct use. not reported	2
	*Papaver somniferum* L. (BCN24941)	Cascall	Analgesic. sedative	Flower. fruit. seed	Collutorium. direct use. not reported. tisane	10
Pinaceae	*Pinus halepensis* Mill. (BCN113592)	Pi. Pi blanc. Pi bord. Pi de pinya llarga. Pi petit. Pinya (fructification)	Antibronchitic. anticatarrhal. antipneumonic. antitussive. expectorant. for abscesses. not reported. vulnerary^a^	Aerial part. flower. fruit. gum/resin. leaf. pollen	Decoction. fumigation. liniment. lotion. not reported. syrup. tisane	33
	*Pinus pinaster* Ait. (BCN36559)	Pi bord. Pi melis	Antibronchitic. antirheumatic	Fruit	Decoction. syrup	2
	*Pinus pinea* L. (BCN26751)	Pi. Pi de llei. Pi de pinya. Pi pinyer	Antibronchitic	Fruit. leaf	Aerosol. syrup	2
	*Pinus* sp.	Pi. Trementina (elaborated product)	Anti-ecchymotic	Gum/resin	Not reported	1
Plantaginaceae	*Plantago lanceolata* L. (BCN32138)	Plantatge de fulla estreta. Plantatge estret	Gingival antiseptic	Leaf	Collutorium	2
	*Plantago major* L. (BCN29910)	Plantatge. Plantatge ample. Plantatge de fulla ampla	Buccal antiseptic. external antiseptic. for amygdalitis. gingival antiseptic. not reported. vulvar anti-inflammatory	Aerial part. leaf	Bath. collutorium. gargle. not reported	8
Poaceae	*Arundo donax* L. (BCN29825)	Canya. Canya americana. Canyer	For trauma	Stem	Direct use	1
	*Phleum phleoides* (L.) Karsten (BCN113804)	Herba de les pedres	Analgesic	Inflorescence	Tisane	1
	*Triticum aestivum* L. (BCN29963)	Blat. Farina (elaborated product). Pa (elaborated product). Palla (elaborated product). Segó (bran)	Antidiarrhoeal^a^. antihelminthic. internal antiseptic^a^. postpartum coadjuvant^a^. restorative^a^	Bran. fruit	Direct use. emulsion. poultice. solution	14
	*Zea mays* L. (BCN29830)	Blat de morassa. Blat de moret. Blat de moro. Farro (elaborated product)	Diuretic. renal anti-inflammatory.. renal lithotriptic. urinary antiseptic	Styles and stigmas	Tisane	20
Ranunculaceae	*Anemone hepatica* L. (BCN29834)	Herba fetgera	For undetermined illnesses^a^, hepatoprotective^b^	Flower. leaf	Direct use. not reported. tisane	11
	*Clematis flammula* L. (BCN29856)	Viadella. Virobella	For warts. not reported	Leaf	Direct use. poultice	3
Rosaceae	*Agrimonia eupatoria* L. (BCN-E-193)	Herba cuquera	Antihelminthic	Flower	Tisane	2
	*Crataegus monogyna* Jacq. (BCN29858)	Arç. Arç blanc	Antihypertensive. cardiotonic	Flower	Tisane	3
	*Cydonia oblonga* Mill. (BCN46849)	Codony (fruit). Codonyat (elaborated product). Codonyer	Antidiarrhoeal. antitussive. not reported. stomachic	Fruit	Alcoholic tincture, decoction, not reported. syrup	15
	*Potentilla reptans* L. (BCN29754)	Gram negre	Antihypertensive	Root	Tisane	1
	*Prunus avium* (L.) L. (BCN29827)	Cirera (fruit). Cirerer	Diuretic, for the influenza	Stem	Tisane	2
	*Pyrus malus* L. subsp. *mitis* (Wallr.) O.Bolòs et J.Vigo (BCN46830)	Poma (fruit). Poma aspra (fruit). Poma cambusina (fruit). Poma camosa (fruit). Poma capçana (fruit). Poma del ciri (fruit). Poma del ciri groga (fruit). Poma del ciri vermella (fruit). Poma *golden* (fruit). Poma rodona (fruit). Poma *royal* (fruit). Pomer. Pomer del ciri. Pomera. Pomera del ciri	Anticatarrhal. restorative	Fruit	Direct use	3
	*Rosa canina* L. (BCN29772)	Rosa. Rosa de pastor. Roser	Anticatarrhal	Fruit	Tisane	1
	*Rubus ulmifolius* Schott (BCN29938)	Bardissa. Mora (fruit). Mora negra (fruit). Romeguera	Antidiarrhoeal. for stings. pharyngeal anti-inflammatory. vulnerary	Leaf. young shoot	Direct use. gargle	4
	*Sanguisorba minor* Scop. (BCN113728)	Esparcet bord	Antdiarrhoeal	Aerial part	Tisane	1
Rubiaceae	*Asperula cynanchica* L. (BCN29634)	Herba prima	Diuretic. intestinal anti-inflammatory. renal lithotriptic. urinary antiseptic	Aerial part	Tisane	16
	*Coffea arabica* L. (BCN46852)^c^	Cafè	Antihypotensive	Seed	Tisane	2
Rutaceae	*Citrus limon* (L.) Burm. (BCN46853)	Llimona (fruit). Llimoner	Anticatarrhal. anti-eccymotic. antihypertensive. digestive. for amygdalitis. restorative	Fruit	Direct use. gargle. not reported. tisane	9
	*Citrus sinensis* (L.) Osbeck (BCN24752)	Taronger. Taronger dolç. Taronja (fruit)	Anticatarrhal	Fruit	Direct use	1
	*Ruta chalepensis* L. (BCN29940)	Ruda	Abortive^b^. analgesic^b^. antihelminthic^b^. anti-inflammatory/antiseptic/cicatrizing mucronal^a^. diuretic, for amygdalitis. for respiratory disorders. laxative^b^. not reported. ocular antiseptic. ruminant antistatic^a^, stomachic	Aerial part	Bath. direct use. liniment. poultice. not reported. tisane	38
Smilacaceae	*Smilax aspera* L. (BCN29951)	Arítjol	Analgesic. antihypertensive	Root	Decoction. liniment	2
Solanaceae	*Nicotiana tabacum* L. (BCN48711)	Tabac	Antihelminthic. antitussive	Leaf	Direct use. fumigation	3
	*Solanum melongena* L. (BCN25004)	Albergínia	For warts	Fruit	Direct use	1
	*Solanum tuberosum* L. (BCN29797)	Patata. Patatera. Trumfera	Antipyrotic	Tuber	Direct use	1
Thymelaeaceae	*Daphne gnidium* L. (BCN29687)	–	Antidiarrhoeal^a^	Aerial part	Direct use	1
Ulmaceae	*Ulmus minor* Mill. (BCN113729)	Om	Anticholesterolemic. antipyrotic	Bark. leaf	Bath. tisane	3
Urticaceae	*Parietaria officinalis* L. subsp. *judaica* (L.) Béguinot (BCN113715)	Blet de paret. Mollerosa	Analgesic. anticatarrhal. antihaemorrhoidal. buccal antiseptic. digestive. for digestive disorders. for stings. for urticaria. postpartum coadjuvant. urinary antiseptic. vaginal antiseptic	Aerial part	Bath. collutorium. direct use. not reported. poultice. tisane	15
	*Urtica dioica* L. (BCN29814)	Ortiga	Against prurigo. antieritematous. antihypertensive. emmenagogue. hemathocathartic. not reported. vasotonic	Aerial part. root	Bath. decoction. direct use. not reported. tisane	10
	*Urtica urens* L. (BCN29966)	Ortiga de fulla petita	Anticatarrhal	Aerial part	Tisane	1
Verbenaceae	*Lippia triphylla* (L’Hér.) O. Kuntze (BCN29886)	Marialluïsa	Digestive. emmenagogue. for headache. laxative. not reported. postpartum coadjuvant^a^. stomachic	Leaf	Decoction. emulsion. tisane	21
Violaceae	*Viola alba* Besser (BCN27286)	Viola. Violeta	For the influenza	Flower	Tisane	1
Vitaceae	*Vitis vinifera* L. (BCN29972)	Raïm (fruit). Sarment. Vi (elaborated product). Vinagre (elaborated product). Vinya	Analgesic. anticholesterolemic. antieritematous. antipyretic. antitussive^a^. for abscesses. for blood disorders. for stings. partum coadjuvant^a^	Fruit. leaf	Bath. collutorium. direct use. medicinal wine. poultice. tisane	14

^a^Ethnoveterinary

^b^Human medicine and ethnoveterinary. No superscripted letter: human medicine

^c^In our country. most coffee industrial presentations are based on *C*. *arabica*. the other taxa. such as *C*. *canephora* Pierre ex A.Froehner and *C*. *liberica* Hiern being clearly minority

The 20 most cited species are included in Table 3. *Sambucus nigra* and *Thymus vulgaris*, with 81 and 78 UR, respectively, are the species heading the ranking. These taxa are among the most cited in other Catalan territories [6, 10, 12, 53, 58]. Concerning the families, Lamiaceae (164 UR; 17.28%) and Adoxaceae (81 UR; 8.54%) are the most reported ones followed by Asteraceae (79 UR; 8.32%), Rutaceae (48 UR; 5.06%), and Oleaceae (41 UR; 4.32%). Lamiaceae and Asteraceae have a high number of representatives in the Mediterranean flora and Rutaceae include the citrus fruit species, whereas Adoxaceae is among the most cited families because of the medicinal importance of *Sambucus nigra*. Although *Olea europaea*, the most relevant species of the Oleaceae, presents medicinal uses as antihypertensive and antihelminthic among others, its high number of use reports is mainly due to the properties of its fruit’s oil as an excipient. One of the 20 top medicinal plants, *Hypericum perforatum*, is illustrated, prepared for use, in Fig. 2.

**Table 3:** List of the 20 most cited species, representing 61.12% of use reports

Taxon	Herbarium voucher	Family	UR	%
*Sambucus nigra* L.	BCN113595	Adoxaceae	81	8.54
*Thymus vulgaris* L.	BCN113590	Lamiaceae	78	8.22
*Matricaria recutita* L.	BCN113594	Asteraceae	46	4.85
*Olea europaea* L.	BCN29898	Oleaceae	41	4.32
*Ruta chalepensis* L.	BCN29940	Rutaceae	38	4.00
*Pinus halepensis* Mill.	BCN113592	Pinaceae	33	3.48
*Hypericum perforatum* L.	BCN113597	Hypericaceae	32	3.37
*Allium sativum* L.	BCN29832	Amaryllidaceae	29	3.06
*Mentha pulegium* L.	BCN113598	Lamiaceae	24	2.53
*Lippia triphylla* (L’Hér.) Kuntze	BCN29886	Verbenaceae	21	2.21
*Zea mays* L.	BCN29830	Poaceae	20	2.11
*Eucalyptus globulus* Labill.	BCN29696	Myrtaceae	18	1.90
*Asperula cynanchica* L.	BCN29634	Rubiaceae	16	1.69
*Cydonia oblonga* Mill.	BCN46849	Rosaceae	15	1.58
*Foeniculum vulgare* Mill.	BCN26350	Apiaceae	15	1.58
*Linum usitatissimum* L.	BCN47281	Linaceae	15	1.58
*Parietaria officinalis* L.	BCN113715	Urticaceae	15	1.58
*Ramonda myconi* (L.) Rchb.	BCN46088	Gesneriaceae	15	1.58
*Rosmarinus officinalis* L.	BCN113599	Lamiaceae	14	1.48
*Triticum aestivum* L.	BCN29963	Poaceae	14	1.48

Our informants referred 50 (36.5%) allocthonous taxa with medicinal uses. This high percentage of allocthonous taxa in the western Gironès is an evidence of both the acculturation process and global market influence. About two-thirds (32) of allochthonous plants recorded are archaeophytes, and only ca. one-third are neophytes (15) or plants not present in the territory and purchased through commerce (three). This nuances the effect of globalization, which is, nevertheless, significant, with 18 out of 137 taxa (13.1%) having been introduced in relatively recent times. In addition, some taxa that could be classified as archaeophytes, since their expansion is not related to the Columbian exchange [67], have been recently introduced, in fact, in popular medicine. This is the case of *Aloe vera* (and other species of the genus used for similar purposes), which could have been introduced as ornamental (and in some cases escaped to the wild) in rather remote times, but are used as medicinal only in recent times; in some cases surely by direct essay in plants cultivated as ornamental and in other cases probably influenced by commercial products based on these plants. In any case, the presence of so-called exotic (allochthonous) plants in European folk medicine would undoubtedly be a good subject for further studies.

The results from the most reported parts are concordant with other Catalan language areas [5, 6, 12], the aerial part being the most cited (306 UR; 32.24%), including young aerial, sterile aerial, flowering aerial, and fructified aerial parts; followed by flowers and inflorescences (213; 22.44%), and fronds or leaves (147; 15.49%).

**Fig. 3:**
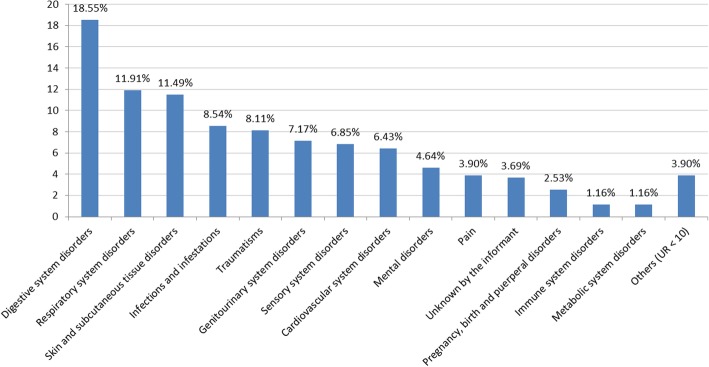
Organic systems disorders, in percentage, quoted in Gironès

A total of 101 types of medicinal uses have been compiled, in which anticatarrhal (59 UR; 6.22%) and stomachic (58; 6.11%) are the most frequent. Conversely, grouped by organic systems disorders, the first positions are exchanged, i.e., digestive system disorders occupy the first position, followed by respiratory system disorders (Fig. 3). The seven first histograms in this figure, altogether accounting for almost three-quarters (73.2%) of medicinal uses, basically represent the kind of remedies mostly used in pharmaceutical ethnobotany or folk medicine and in phytotherapy in general, importantly focused on chronic and usually not very severe illnesses [50, 68, 69]. This does not at all preclude ethnobotany from being a solid basis for drug development, as already stated by Chadwick & Marsh [70] and recently confirmed, for instance, with the application of artemisinin and derivatives as the most important antimalarial drugs [71]. In this respect, some of the uses recorded in the present study, apart from those addressing mild ailments—which are also important for everyday life—could be worthy of further research related to the development of drugs against cardiovascular, mental or immune system disorders, or as a cancer preventive.

The mode of preparation is not very variable. With just two preparation forms, we collect almost 50% of reports. Tisane, including infusion and decoction, is the most commonly used form, reported 324 times, and represents 34.14% of forms, followed by remedies without pharmaceutical form (146; 15.38%).

The number of medicinal plants per informant (MP/I) is 2.40, comparable to the results obtained in Castelló and Ripollès, but lower than in Guilleries (Table 1). This is due to the fact that the number of medicinal plants cited does not increase proportionally to the number of informants but, as the sample grows, the citation of new species becomes less and less frequent. In the same direction, the average of citations for plants does not grow when we increase the number of informants, but tends to stabilize [72].

The number of medicinal plants per inhabitant is slightly higher value than neighboring areas such as Cerdanya or Ripollès and lower than Alt Empordà (Table 1). The number of medicinal plants per km^2^ (MP/km^2^) is 0.73, much bigger than those calculated in Cerdanya, Alt Empordà, les Guilleries, Ripollès, and Montseny. We believe that these data are useful to compare territories, but it has to be taken into account that they do not basically depend on the informant sampling but on the total population or extension of each studied area.

This study has also inventoried 55 plant mixtures with 80 different taxa; the 3 most reported species are *Thymus vulgaris* with 16 citations and present in a 29.09% of mixtures, *Triticum aestivum* (14; 25.45%) and *Rosmarinus officinalis* (13; 23.64%). These mixtures were used to treat 28 diseases, and if we group the diseases by organic system disorders, respiratory, and pregnancy, birth and puerperal disorders are ranked in first position, both with 20% of mixtures. The first disorder is associated with human use and the second one with veterinary use as a postpartum coadjuvant, results that in accordance with those mentioned in Gras et al. [52]. The number of species per mixture varies largely from 1 mixture to another, ranging from 2 to 28 species per mixture and the average number of taxa or ingredients is 3.31. The mixture with the highest number of taxa is a poultice for pneumonia treatment.

The informant consensus factor (F_IC_) for mixtures data is 0.56, a lower value than in the recent before-mentioned study, devoted to plant mixtures in two Catalan territories [52]. The index of taxon usefulness in mixtures (ITUM), calculated for all taxa with more than three use reports in mixtures, is lower than 0.50 in almost all cases, except in *Triticum aestivum* (0.50) and *Lavandula stoechas* (0.67). The results show that there is not a trend of exclusiveness of taxa use in mixtures, but taxa used in mixtures are also employed alone.

### Food uses

Food uses are the most reported by the informants in this area: 224 taxa belonging to 66 families constitute an important dataset, accumulating 1933 use reports. Six of these taxa have only been determined at the genus level, and from the remaining 218 ones, 3 of them have infraspecific categories. All of these results show a clear difference with other studied areas, where the medicinal uses predominate over food uses [6, 10, 58]. We do not have a clear hypothesis that explains this surprising result, but although some biases could have occurred in the interviewing process, the importance of crops and the proximity of the markets are two factors that need to be taken into account in this area.

Concerning the destination, a 70.67% of these taxa are used in human food, a 28.30% in animal feed, and there is no information for the remaining 1.03%. The mean of food taxa cited by informant is 3.42 for human food and 1.42 for animal feed.

The most reported families for human food consumption (Table 4) are Lamiaceae (272 UR), Rosaceae (174 UR), and Asteraceae (126 UR). These are families well represented in the Mediterranean flora as in the case of medicinal plants, and some of them comprising relevant categories for human food such as aromatic plants and fruit trees. The rare families concerning both the number of species and number of use reports usually correspond to taxa acquired through commerce. The five species that have received more citations are *Mentha spicata* (50 UR), *Reichardia picroides* (37 UR), *Laurus nobilis* (36 UR), *Arbutus unedo* (35 UR), and *Rubus ulmifolius* (34 UR). These are wild species, but *Laurus nobilis* is also very frequently cultivated near houses. Additionally, *Reichardia picroides*, one of the most well-known food (and also feed) plants in the Catalan linguistic area [34, 58] and in other Mediterranean areas [32, 73], is a wild species, which, as reported by Maggini et al. [74] in a study in Tuscany involving cultivation of several ecotypes, seems to be a promising vegetable crop, with nutraceutical properties. Further research in this sense in different areas would be desirable to confirm this point.

**Table 4:** Food plants reported in the studied area

Family	Taxon (voucher)	Catalan vernacular names	Preparation	Used part	Wild (W)/cultivated (C)	Use reports
Adoxaceae	*Sambucus nigra* L. (BCN113595)	Sabuquer. Saüc. Saüquer	Alcoholic beverage made with wine. Cooked in oil. Cooked in wine. Cooked with sugar. High-grade alcoholic beverage	Fruit. Inflorescence	W	15
Amaranthaceae	*Beta vulgaris* L. subsp. *vulgaris* var. *crassa* (Alef.) Helm (BCN50761)	Bleda. Polpa (elaborated product). Remolatxa. Sucre (elaborated product). Sucre candi (elaborated product)	Air dried. Alcoholic beverage made with wine. High-grade alcoholic beverage	Root	C	9
Amaryllidaceae	*Allium cepa* L. (BCN28655)	Ceba	Boiled in water. Cooked. Raw	Bulb	C	4
	*Allium porrum* L. (BCN28791)	Porro	Condiment	Leaf	C	2
	*Allium sativum* L. (BCN29832)	All	Boiled in water. Condiment. Cooked in oil. Raw	Bulb	C	10
Apiaceae	*Apium nodiflorum* (L.) Lag. (BCN113736)	Créixec. Creixen	Raw	Aerial part	W	4
	*Carum carvi* L. (BCN29642)	Comí	High-grade alcoholic beverage	Flower	W	1
	*Cuminum cyminum* L. (BCN-E-194)	Comí	High-grade alcoholic beverage	Seed	W	1
	*Daucus carota* L. subsp. *sativus* (Hoffm.) Arcang. (BCN46847)	Pastanaga	Boiled in water	Root	C	1
	*Eryngium campestre* L. (BCN31274)	Espinacal	High-grade alcoholic beverage	Flower	W	1
	*Foeniculum vulgare* Mill. (BCN26350)	Fonoll	Boiled in water. Condiment. High-grade alcoholic beverage	Aerial part. Inflorescence	W	27
	*Petroselinum crispum* (Mill.) Hill (BCN29905)	Julivert	Condiment	Leaf	C	4
	*Pimpinella anisum* L. (BCN47278)	Anís verd. Matafaluga	Condiment. High-grade alcoholic beverage	Fruit	C	6
Apocynaceae	*Vinca major* L. (BCN25039)	Vinca. Vincapervinca	High-grade alcoholic beverage	Flower	W	2
Asparagaceae	*Aphyllanthes monspeliensis* L. (BCN29627)	Llonsa. Pa de cucut	Raw	Aerial part. Flower	W	3
	*Asparagus acutifolius* L. (BCN29976)	Espàrgol. Esparreguera. Espàrrec	Cooked in oil. High-grade alcoholic beverage	Leaf. Young shoot	W	12
Aspleniaceae	*Asplenium adiantum-nigrum* L. (BCN113596)	Fulleta	High-grade alcoholic beverage	Frond	W	1
Asteraceae	*Achillea ageratum* L. (BCN113701)	Herba del fàstic	High-grade alcoholic beverage	Inflorescence	W	1
	*Achillea millefolium* L. (BCN113708)	Cordonet. Herba de les milfulles	High-grade alcoholic beverage	Aerial part	W	1
	*Arctium minus* Bernh. (BCN113727)	–	Boiled in water	Stem	W	1
	*Artemisia abrotanum* L. (BCN31263)	Broida	Condiment	Leaf	W	1
	*Artemisia absinthium* L. (BCN29837)	Artemisa. Donzell	High-grade alcoholic beverage	Aerial part	W	1
	*Bellis perennis* L. (BCN31264)	Margaridoia	Raw	Leaf	W	2
	*Calendula officinalis* L. (BCN29977)	Calèndula	High-grade alcoholic beverage. Raw	Inflorescence	C	2
	*Centaurea aspera* L. (BCN113579)	Caps de burro. Flor del sucre. Travalera	High-grade alcoholic beverage	Inflorescence	W	2
	*Chondrilla juncea* L. (BCN29852)	Màstec	Preserved in vinegar. Raw	Leaf	W	29
	*Cichorium endivia* L. (BCN46854)	Escarola	Condiment	Leaf	C	1
	*Cichorium intybus* L. (BCN29660)	Cama-roja. Xicoia. Xicoina. Xicòria	Raw	Leaf	W	6
	*Cynara cardunculus* L. (BCN-E-192)	Herba presonera. Presó. Presonera. Presora	Condiment	Inflorescence	C	18
	*Helichrysum stoechas* (L.) Moench (BCN29872)	Mançanilla. Sempreviva	High-grade alcoholic beverage	Aerial part. Inflorescence	W	3
	*Matricaria recutita* L. (BCN113594)	Camamilla. Camamilla romana	High-grade alcoholic beverage	Aerial part. Inflorescence	W	11
	*Pallenis spinosa* (L.) Cass. (BCN31291)	Mala mare	High-grade alcoholic beverage	Inflorescence	W	2
	*Reichardia picroides* (L.) Roth (BCN113704)	Cosconilla	High-grade alcoholic beverage. Raw	Leaf	W	37
	*Santolina chamaecyparissus* L. (BCN113709)	Espernallac. Santolina	High-grade alcoholic beverage. Milk-based beverage	Inflorescence	W	2
	*Silybum marianum* (L.) Gaertn. (BCN29958)	Card	Boiled in water	Stem	W	1
	*Sonchus* sp.	Llepsó. Lletissó. Llistó	Raw	Aerial part	W	1
	*Stevia rebaudiana* (Bertoni) Bertoni (BCN30644)	Estèvia	Not reported	Leaf	C	1
	*Taraxacum officinale* Weber in Wiggers (BCN25948)	Dent de lleó. Xicoia	Beverage made with water. High-grade alcoholic beverage. Raw	Leaf. Not reported. Root	W	3
Betulaceae	*Corylus avellana* L. (BCN29831)	Avellana (fruit). Avellana del queixal (fruit). Avellaner. Avellaner negret	Cooked. Raw	Fruit	W	7
Brassicaceae	*Brassica napus* L. (BCN46856)	Nap. Nap de bou. Nap del camp	Boiled in water	Root	C	1
	*Brassica oleracea* L. subsp. *oleracea* (BCN32181)	Bròquil. Col. Col aloma	Preserved in salt and water	Leaf	C	1
	*Capsella bursa-pastoris* (L.) Medic. (BCN46079)	Bossa de pastor. Caps blancs	High-grade alcoholic beverage. Raw	Aerial part	W	2
	*Raphanus raphanistrum* L. subsp. *sativus* (L.) Domin (BCN49860)	Rave	Raw	Stem	C	1
	*Rorippa nasturtium-aquaticum* (L.) Hayek subsp. *nasturtium-aquaticum* (BCN29771)	Créixec. Creixen	Raw	Aerial part	W	6
Cactaceae	*Opuntia maxima* Mill. (BCN46078)	Figuera de moro	Condiment	Leaf	C	1
Campanulaceae	*Campanula rapunculus* L. (BCN50763)	Repunxó	Raw	Leaf. Root. Whole plant	W	22
Cannabaceae	*Celtis australis* L. (BCN29845)	Lledó (fruit). Lledoner	High-grade alcoholic beverage. Raw	Fruit	C	15
Caprifoliaceae	*Lonicera implexa* Ait. (BCN113802)	Lligabosc. Mareselva. Xuclamel	High-grade alcoholic beverage. Raw	Aerial part. Flower	W	13
	*Scabiosa atropurpurea* L. (BCN29947)	Escabiosa	High-grade alcoholic beverage	Flower	W	5
	*Valerianella locusta* (L.) Laterrade (BCN49861)	Margarideta. Margaridoia. Marieta	Raw	Leaf	w	5
Caryophyllaceae	*Dianthus caryophyllus* L. (BCN31272)	Clavell. Clavell domèstic	High-grade alcoholic beverage	Flower	C	4
	*Dianthus seguieri* Vill. (BCN113734)	Clavell de pastor	High-grade alcoholic beverage	Flower	W	1
	*Herniaria glabra* L. (BCN113577)	Herba de les mil granes. Mil granes	High-grade alcoholic beverage	Aerial part	W	5
	*Silene vulgaris* (Moench) Garcke (BCN29948)	Culivells	Boiled in water	Leaf	W	5
Cistaceae	*Cistus salviifolius* L. (BCN36767)	Estepa. Mòdega	High-grade alcoholic beverage	Flower	W	2
Clusiaceae	*Hypericum perforatum* L. (BCN113597)	Flor de Sant Joan. Herba de cop. Herba de Sant Joan	High-grade alcoholic beverage	Aerial part. Flower	W	12
Convolvulaceae	*Convolvulus arvensis* L. (BCN29663)	Corretjola	High-grade alcoholic beverage	Flower	W	2
Crassulaceae	*Sempervivum tectorum* L. (BCN26780)	Matifoc	High-grade alcoholic beverage	Leaf	W	1
Cucurbitaceae	*Citrullus lanatus* (Thunb.) Matsumara et Nakai (BCN29662)	Síndria. Xíndria	Cooked with sugar	Epicarp. Fruit	C	2
	*Cucumis melo* L. (BCN46851)	Meló (fruit)	Cooked with sugar. Raw	Fruit	C	6
	*Cucurbita ficifolia* C.D.Bouché in Verh. (BCN29980)	Carabassa de cabell d’àngel	Cooked with sugar	Fruit	C	1
	*Cucurbita pepo* L. var. *oblonga* Link (BCN29859)	Carabassó (fruit)	Boiled in water. Cooked in oil	Flower. Fruit	C	4
	*Cucurbita pepo* L. var. *pepo* (BCN49858)	Carbassa (fruit). Carbassera. Rabequet (fruit)	Boiled in water	Fruit	C	2
Cupressaceae	*Juniperus communis* L. (BCN113589)	Ginebre. Ginebró	Air dried. Alcoholic beverage made with wine. Condiment. High-grade alcoholic beverage	Fruit	W	12
	*Juniperus oxycedrus* L. (BCN29879)	Càdec	High-grade alcoholic beverage	Fruit	W	1
Equisetaceae	*Equisetum arvense* L. (BCN24767)	Cua de cavall. Sangnua	High-grade alcoholic beverage	Aerial part	W	1
	*Equisetum* sp.	Cua de cavall. Sangnua	High-grade alcoholic beverage	Aerial part	W	4
	*Equisetum telmateia* Ehrh. (BCN113581)	Cua de cavall. Sangnua	High-grade alcoholic beverage	Aerial part	W	3
Ericaceae	*Arbutus unedo* L. (BCN29836)	Arboç. Bola d’arboç (fruit). Cirera d’arboç (fruit). Cirerer d’arboç	Cooked with sugar. High-grade alcoholic beverage. Raw	Fruit	W	35
	*Calluna vulgaris* (L.) Hull (BCN113722)	Bronsa. Bronsó	Raw	Flower	W	1
	*Erica arborea* L. (BCN113593)	Bruc. Bruc boal. Bruc bord. Bruc d’ull de bou. Bruc de bou. Bruc de llei	High-grade alcoholic beverage	Flower	W	3
Euphorbiaceae	*Euphorbia* sp.	Llet de Tereses. Lletdetresa	Condiment	Latex	W	1
Fabaceae	*Acacia dealbata* Link. (BCN29973)	Mimosa	High-grade alcoholic beverage	Flower	C	2
	*Ceratonia siliqua* L. (BCN32177)	Garrofa (fruit)	Air dried	Fruit	C	5
	*Glycyrrhiza glabra* L. (BCN47276)	Regalíssia	High-grade alcoholic beverage	Root	W	2
	*Lathyrus latifolius* L. (BCN29712)	Pèsol bord	Boiled with water. High-grade alcoholic beverage	Flower. Fruit	W	4
	*Onobrychis viciifolia* Scop. (BCN113732)	Esparcet. Trepadella	High-grade alcoholic beverage	Flower	C	2
	*Phaseolus vulgaris* L. (BCN46837)	Fesol de l’ull *marrón*. Fesol de l’ull negre. Fesol de Santa Pau. Fesol del bitxet gros. Fesol de bitxet petit. Fesol menut. Fesola. Mongeta	Boiled in water	Seed	C	5
	*Robinia pseudoacacia* L. (BCN31298)	Acàcia. Acàcia de jardí	High-grade alcoholic beverage	Flower	C	2
	*Spartium junceum* L. (BCN29956)	Ginesta	High-grade alcoholic beverage	Flower	W	9
	*Trifolium incarnatum* L. (BCN25026)	Fenc	Boiled in water. High-grade alcoholic beverage	Aerial part. Flower	C	3
	*Vicia faba* L. (BCN46826)	Fava	Boiled in water. Cooked. Cooked in oil	Seed	C	6
	*Vicia sativa* L. (BCN47746)	Veça	Raw	Aerial part	C	1
Fagaceae	*Castanea sativa* Mill. (BCN29844)	Castanya (fruit). Castanyer	Boiled in water. Cooked	Fruit	C	9
	*Quercus ilex* L. (BCN113730)	Aglà (fruit). Alzina. Aulina. Gla (fruit)	Cooked. High-grade alcoholic beverage	Flower. Fruit	W	7
	*Quercus suber* L. (BCN46829)	Suro. Alzina surera	Air dried	Fruit	W	1
Gesneriaceae	*Ramonda myconi* (L.) Reichenb. (BCN46088)	Orella d’os	High-grade alcoholic beverage	Aerial part. Leaf	W	4
Hydrangeaceae	*Philadelphus coronarius* L. (BCN27261)	Xeringuilla	High-grade alcoholic beverage	Flower	C	2
Iridaceae	*Crocus sativus* L. (BCN32170)	Safrà	Condiment	Styles and stigmas	C	1
Juglandaceae	*Juglans regia* L. (BCN29877)	Noguer. Nou (fruit). Nou verda (fruit)	Air dried. Alcoholic beverage made with wine. Condiment. High-grade alcoholic beverage	Fruit. Leaf	C	29
Lamiaceae	*Hyssopus officinalis* L. (BCN29709)	Hisop	High-grade alcoholic beverage	Aerial part. Flower	W	5
	*Lamium flexuosum* Ten. (BCN26731)	Ortiga blanca	High-grade alcoholic beverage	Flower	W	2
	*Lavandula dentata* L. (BCN29715)	Lavanda	High-grade alcoholic beverage	Flower	W	1
	*Lavandula latifolia* Medic. (BCN113740)	Barballó. Espígol. Lavanda	High-grade alcoholic beverage	Aerial part	W	4
	*Lavandula stoechas* L. (BCN113714)	Cap d’ase. Tomanyí	High-grade alcoholic beverage	Aerial part. Flower	W	6
	*Melissa officinalis* L. (BCN113713)	Melissa. Tarongina	High-grade alcoholic beverage	Aerial part. Leaf	C	12
	*Mentha* ×*piperita* L. (BCN113813)	Menta. Menta de la xocolata. Menta piperita. Menta romana	Condiment. High-grade alcoholic beverage	Aerial part	C	15
	*Mentha pulegium* L. (BCN113598)	Poliol. Poniol	High-grade alcoholic beverage	Aerial part. Flower	W	8
	*Mentha spicata* L. (BCN113812)	Menta. Menta de la sopa. Menta silvestre. Menta espicata. Menta verdadera	Boiled with water. Condiment. High-grade alcoholic beverage	Aerial part	W	50
	*Mentha suaveolens* Ehrh. (BCN113810)	Menta blanca	High-grade alcoholic beverage	Aerial part	W	3
	*Nepeta cataria* L. (BCN113798)	Gatera. Nepta	High-grade alcoholic beverage	Aerial part	W	3
	*Ocimum basilicum* L. (BCN29897)	Alfàbrega	High-grade alcoholic beverage	Aerial part	C	6
	*Origanum majorana* L. (BCN113585)	Marduix	Condiment. High-grade alcoholic beverage	Aerial part. Flower	C	27
	*Origanum vulgare* L. (BCN113705)	Orenga	Condiment. High-grade alcoholic beverage. Not reported	Aerial part	W	18
	*Prunella grandiflora* (L.) Scholler (BCN24956)	Herba del traïdor	High-grade alcoholic beverage	Flower	W	1
	*Prunella laciniata* (L.) L. (BCN29481)	Herba del traïdor	High-grade alcoholic beverage	Flower	W	1
	*Prunella vulgaris* L. (BCN113578)	Herba del traïdor	High-grade alcoholic beverage	Flower	W	2
	*Rosmarinus officinalis* L. (BCN113599)	Romaní	Condiment. High-grade alcoholic beverage. Raw	Aerial part. Flower	W	27
	*Salvia microphylla* Humb., Bonpl. & Kunth (BCN113718)	Menta. Menta americana. Menta romana	High-grade alcoholic beverage	Aerial part	C	4
	*Salvia officinalis* L. subsp. *lavandulifolia* (Vahl) Gams (BCN29780)	Sàlvia de fulla estreta	High-grade alcoholic beverage	Aerial part	W	1
	*Salvia officinalis* L. subsp. *officinalis* (BCN113583)	Sàlvia. Sàlvia de fulla ampla	Condiment. High-grade alcoholic beverage	Aerial part. Flower. Leaf	C	11
	*Satureja montana* L. (BCN113741)	Sajolida	Condiment. High-grade alcoholic beverage	Aerial part	W	12
	*Sideritis hirsuta* L. (BCN113582)	Herba de Sant Antoni	High-grade alcoholic beverage	Aerial part	W	1
	*Stachys byzantina* C. Koch (BCN113707)	Fulles de la mare de Déu. Planta de vellut	High-grade alcoholic beverage	Leaf	C	3
	*Stachys officinalis* (L.) Trevisan (BCN25011)	Brotònica	High-grade alcoholic beverage	Flower	C	2
	*Teucrium chamaedrys* L. (BCN29806)	Brotònica	High-grade alcoholic beverage	Aerial part	W	1
	*Thymus* ×*citriodorus* (Pers.) Schreber (BCN113803)	Farigola de xocolata. Farigola llimonera	High-grade alcoholic beverage	Aerial part	C	1
	*Thymus serpyllum* L. (BCN113719)	Farigola de pastor. Farigoleta. Salsa de pastor	Boiled in water. Condiment. High-grade alcoholic beverage	Aerial part	W	11
	*Thymus vulgaris* L. (BCN113590)	Farigola	Condiment. High-grade alcoholic beverage	Aerial part	W	34
Lauraceae	*Cinnamomum zeylanicum* Nees (BCN47283)	Canyella	Condiment. High-grade alcoholic beverage	Bark	C	10
	*Laurus nobilis* L. (BCN113717)	Llord. Llorer	Condiment. High-grade alcoholic beverage	Aerial part. Leaf	C	36
Liliaceae	*Lilium candidum* L. (BCN46841)	Lliri de Sant Antoni. Lliri de Sant Josep	High-grade alcoholic beverage	Flower	C	4
Lythraceae	*Punica granatum* L. (BCN29764)	Magrana (fruit). Magraner. Magraner agre. Magraner bord. Magraner dolç	Cooked with sugar. High-grade alcoholic beverage. Raw	Flower. Fruit	C	6
Magnoliaceae	*Magnolia grandiflora* L. (BCN64396)	Magnòlia	High-grade alcoholic beverage	Flower	C	2
Malvaceae	*Althaea officinalis* L. (BCN113799)	Malví	High-grade alcoholic beverage	Root	W	1
	*Malva sylvestris* L. (BCN29889)	Malva. Malva rosa	High-grade alcoholic beverage	Flower	W	8
	*Theobroma cacao* L. (BCN30763)	Xocolata (elaborated product)	Condiment	Seed	C	1
	*Tilia cordata* Mill. (BCN26784)	Til·la	High-grade alcoholic beverage	Bract with inflorescence	W	5
	*Tilia platyphyllos* Scop. (BCN113739)	Tei. Til·la. Til·ler de bosc	High-grade alcoholic beverage	Bract with inflorescence	W	7
Moraceae	*Ficus carica* L. (BCN24887)	Figa (infructescence). Figa d’Alacant (infructescence). Figa de coll de senyora (infructescence). Figa de coll llarg blanca (infructescence). Figa de coll llarg negra (infructescence). Figa de pota de cavall (infructescence). Figa de Sant Joan (infructescence). Figa negra (infructescence). Figuera. Figuera de coll de senyora	Air dried. Cooked with sugar. Raw	Infructescence	C	11
Myristicaceae	*Myristica fragrans* Houtt. (BCN50769)	Nou moscada	High-grade alcoholic beverage	Fruit	C	6
Myrtaceae	*Eucalyptus globulus* Labill. (BCN29696)	Eucaliptu. Eucaliptus	High-grade alcoholic beverage	Leaf	C	2
	*Syzygium aromaticum* (L.) Merr. et Perry (BCN47279)	Clau d’espècia. Clau de pot. Clavell. Clavell d’espècia	High-grade alcoholic beverage	Floral bud	C	7
Oleaceae	*Ligustrum vulgare* L. (BCN24915)	Olivereta	High-grade alcoholic beverage	Flower	W	2
	*Olea europaea* L. subsp. *europaea* (BCN29898)	Oli (elaborated product). Oli d’oliva (elaborated product). Olivera. Oliva (fruit)	Condiment. Boiled in water. High-grade alcoholic beverage. Preserved in salt and water. Raw	Aerial part. Flower. Fruit	C	22
	*Syringa vulgaris* L. (BCN29959)	Lilà	High-grade alcoholic beverage	Flower	C	2
Papaveraceae	*Papaver rhoeas* L. (BCN29903)	Gallaret. Pipiripip. Quiquiriquí. Rosella	Boiled in water. High-grade alcoholic beverage. Raw	Aerial part. Leaf	W	4
	*Papaver somniferum* L. (BCN24941)	Cascall	High-grade alcoholic beverage	Aerial part	W	2
Pinaceae	*Pinus halepensis* Mill. (BCN113592)	Pi. Pi blanc. Pi bord. Pi de pinya llarga. Pi petit. Pinya (fructification)	High-grade alcoholic beverage	Flower. Fructification. Young shoot	W	10
	*Pinus pinaster* Ait. (BCN36559)	Pi bord. Pi melis	Air dried. High-grade alcoholic beverage	Flower. Leaf	W	2
	*Pinus pinea* L. (BCN26751)	Pi. Pi de llei. Pi de pinya. Pi pinyer	High-grade alcoholic beverage	Fructification	W	1
Plantaginaceae	*Plantago lanceolata* L. (BCN32138)	Plantatge de fulla estreta. Plantatge estret	High-grade alcoholic beverage	Flower. Leaf	W	4
	*Plantago major* L. (BCN29910)	Plantatge. Plantatge ample. Plantatge de fulla ampla	High-grade alcoholic beverage	Flower. Leaf	W	5
Poaceae	*Avena barbata* Pott ex Link in Schrad. (BCN49867)	Avena. Cugula	High-grade alcoholic beverage	Fruit	W	2
	*Avena sativa* L. (BCN29839)	Civada	High-grade alcoholic beverage	Fruit	C	2
	*Briza media* L. (BCN113733)	Belluguets	High-grade alcoholic beverage	Flower	W	2
	*Hordeum vulgare* L. (BCN46843)	Ordi	Cooked	Fruit	C	1
	*Panicum miliaceum* L. (BCN12911)	Mill	Air dried	Fruit	C	2
	*Saccharum officinarum* L. (BCN50771)	Rom (elaborated product). Sucre roig (elaborated product)	Alcoholic beverage made with wine. High-grade alcoholic beverage	Stem	C	4
	*Secale cereale* L. (BCN46828)	Sègol. Sègal	Boiled in water. Cooked	Fruit	C	7
	*Sorghum bicolor* (L.) Moench (BCN31310)	Melca. Sorgo	Cooked	Fruit	C	1
	*Triticum aestivum* L. (BCN29963)	Blat. Farina (elaborated product). Pa (elaborated product). Palla (elaborated product). Segó (bran)	Air dried. Boiled in water. Cooked. High-grade alcoholic beverage	Bran. Fruit. Spike	C	21
	*Zea mays* L. (BCN29830)	Blat de morassa. Blat de moret. Blat de moro. Farro (elaborated product)	Air dried. Boiled in water. Cooked. High-grade alcoholic beverage	Fruit. Styles and stigmas	C	26
Polygonaceae	*Fagopyrum esculentum* Moench (BCN24886)	Fajol	Boiled in water. Cooked	Seed	C	16
Portulacaceae	*Portulaca oleracea* L. (BCN46835)	Verdolaga	Boiled in water. High-grade alcoholic beverage. Raw	Aerial part	W	23
Ranunculaceae	*Anemone hepatica* L. (BCN29834)	Herba fetgera	High-grade alcoholic beverage	Aerial part. Leaf	W	5
	*Clematis recta* L. (BCN113720)	Viadella	High-grade alcoholic beverage	Flower	W	1
Rhamnaceae	*Ziziphus jujuba* Mill. (BCN113700)	Gínjol (fruit). Ginjoler	Raw	Fruit	C	3
Rosaceae	*Agrimonia eupatoria* L. (BCN-E-193)	Herba cuquera	High-grade alcoholic beverage	Flower	W	2
	*Crataegus monogyna* Jacq. (BCN29858)	Arç. Arç blanc	Cooked in oil. High-grade alcoholic beverage	Flower	W	4
	*Cydonia oblonga* Mill. (BCN46849)	Codony (fruit). Codonyat (elaborated product). Codonyer	Cooked with sugar. High-grade alcoholic beverage	Fruit	C	24
	*Fragaria vesca* L. (BCN29697)	Maduixa (infructescence). Maduixa de bosc (infructescence). Maduixa petita (infructescence). Maduixer. Maduixer de bosc. Maduixeta (infructescence)	Raw	Infructescence	W	16
	*Fragaria viridis* Weston (BCN62767)	Marrans	Raw	Infructescence	C	1
	*Mespilus germanica* L. (BCN50768)	Nespler. Nespla de bosc. Nespra. Nespro	Raw	Fruit	C	9
	*Prunus avium* (L.) L. (BCN29827)	Cirera (fruit). Cirerer	High-grade alcoholic beverage. Raw	Flower. Fruit	C	6
	*Prunus domestica* L. subsp. *domestica* (BCN46834)	Pruna (fruit). Pruner. Pruna clàudia (fruit). Pruna de colló de frare (fruit)	Cooked with sugar. High-grade alcoholic beverage. Raw	Flower. Fruit	C	10
	*Prunus dulcis* (Mill.) Weeb. (BCN46833)	Ametller	High-grade alcoholic beverage	Flower	C	1
	*Prunus persica* (L.) Batsch (BCN46832)	Préssec (fruit). Préssec cardinal (fruit). Préssec de coure (fruit). Préssec duran (fruit). Préssec groc (fruit). Préssec groc d’agost (fruit). Préssec mollar (fruit). Préssec sang de llebre (fruit). Préssec de Sant Joan (fruit). Préssec de Sant Pere (fruit). Presseguer	Cooked with sugar. High-grade alcoholic beverage. Raw	Flower. Fruit	C	9
	*Prunus spinosa* L. (BCN30005)	Aranyó (fruit). Aranyoner. Arç. Arç negre. Arça	Cooked with sugar. High-grade alcoholic beverage. Raw	Fruit	W	13
	*Pyrus communis* L. subsp. *communis* (BCN46831)	Pera (fruit). Pera conference (fruit). Pera de Sant Joan (fruit). Pera rogija (fruit). Perer. Perer mau	Alcoholic beverage made with wine. Cooked with sugar. High-grade alcoholic beverage. Raw	Flower. Fruit	C	8
	*Pyrus malus* L. subsp. *mitis* (Wallr.) O.Bolòs et J.Vigo (BCN46830)	Poma (fruit). Poma aspra (fruit). Poma cambusina (fruit). Poma camosa (fruit). Poma capçana (fruit). Poma del ciri (fruit). Poma del ciri groga (fruit). Poma del ciri vermella (fruit). Poma *golden* (fruit). Poma rodona (fruit). Poma *royal* (fruit). Pomer. Pomer del ciri. Pomera. Pomera del ciri	Cooked with sugar. High-grade alcoholic beverage. Raw	Flower. Fruit	C	24
	*Rosa canina* L. (BCN29772)	Rosa. Rosa de pastor. Roser	High-grade alcoholic beverage	Flower	W	6
	*Rosa* sp.	Rosa. Rosa de jardí. Roser	High-grade alcoholic beverage	Flower. Leaf	C	2
	*Rubus idaeus* L. (BCN29774)	Gerd (fruit)	Cooked with sugar. Raw	Fruit	W	3
	*Rubus ulmifolius* Schott (BCN29938)	Bardissa. Mora (fruit). Mora negra (fruit). Romeguera	Cooked with sugar. High-grade alcoholic beverage. Raw	Flower. Fruit. Young shoot	W	34
	*Sorbus domestica* L. (BCN46827)	Server	Raw	Fruit	C	1
	*Sorbus torminalis* (L.) Crantz (BCN43294)	–	Raw	Fruit	W	1
Rubiaceae	*Asperula cynanchica* L. (BCN29634)	Herbaprima	High-grade alcoholic beverage	Aerial part	W	1
	*Coffea arabica* L. (BCN46852)^a^	Cafè	Beverage made with water. High-grade alcoholic beverage	Seed	C	9
Rutaceae	*Citrus aurantium* L. (BCN46080)	Taronger agre. Taronger amarg. Taronger bord. Taronja agra (fruit)	Condiment. Cooked with sugar. High-grade alcoholic beverage.	Leaf. Fruit	C	4
	*Citrus japonica* Thunb. (BCN113966)	Llimona de Xipre	High-grade alcoholic beverage	Fruit	C	1
	*Citrus limon* (L.) Burm. (BCN46853)	Llimona (fruit). Llimoner	Condiment. High-grade alcoholic beverage. Raw	Epicarp. Fruit. Leaf	C	19
	*Citrus sinensis* (L.) Osbeck (BCN24752)	Taronger. Taronger dolç. Taronja (fruit)	Condiment. Cooked with sugar. High-grade alcoholic beverage	Epicarp. Flower. Fruit	C	15
	*Ruta chalepensis* L. (BCN29940)	Ruda	Condiment. High-grade alcoholic beverage. Raw	Aerial part. Leaf	W	17
Schisandraceae	*Illicium verum* Hook.f. (BCN47282)	Anís estrellat	High-grade alcoholic beverage	Fruit	C	4
Scrophulariaceae	*Verbascum* sp.	Cua de guilla	High-grade alcoholic beverage	Flower	W	1
Solanaceae	*Capsicum annuum* L. (BCN42737)	Bitxo	Cooked in oil. Preserved in salt and water	Fruit	C	12
	*Solanum lycopersicum* L. (BCN29952)	Tomata (fruit). Tomata de guardar (fruit). Tomata de la meta (fruit). Tomata de penjar (fruit). Tomata dels tres *cantos* (fruit). Tomata plena (fruit). Tomata poma (fruit)	Cooked in oil. Preserved in salt and water. Cooked with sugar. Raw	Fruit	C	12
	*Solanum tuberosum* L. (BCN29797)	Patata. Patatera. Trumfera	Boiled in water. Cooked	Tuber	C	3
Ulmaceae	*Ulmus minor* Mill. (BCN113729)	Om	Boiled in water	Leaf	W	1
Urticaceae	*Parietaria officinalis* L. subsp. *judaica* (L.) Béguinot (BCN113715)	Blet de paret. Mollerosa	High-grade alcoholic beverage	Aerial part	W	5
	*Urtica dioica* L. (BCN29814)	Ortiga	Boiled in water	Aerial part	W	4
Verbenaceae	*Lippia triphylla* (L’Hér.) O.Kuntze (BCN29886)	Marialluïsa	High-grade alcoholic beverage	Aerial part. Leaf	C	12
Violaceae	*Viola tricolor* L. (BCN25041)	Pensaments	High-grade alcoholic beverage	Flower	W	2
Vitaceae	*Vitis vinifera* L. (BCN29972)	Raïm (fruit). Sarment. Vi (elaborated product). Vinagre (elaborated product). Vinya	Condiment. Cooked with sugar. Preserved in vinegar. Raw	Fruit	C	12

^a^In our country, most coffee industrial presentations are based on *C*. *arabica*, the other taxa, such as *C*. *canephora* Pierre ex A.Froehner and *C*. *liberica* Hiern being clearly minority

Fruit (including fructification in the gymnosperms) and infructescence are the most used plant parts (29.28%), followed by aerial part, including the whole plant sometimes used in the same way by our informants (27.6%), flower and inflorescence (17.28%), and leaf (14.2%). Regarding the preparation forms, two of them are nearly tied at the top of the ranking; the first one, the beverage prepared with alcohol (39.02%) for the importance of traditional liqueur called *ratafia*, and the second one, the raw plant (20.79%), which is not really a form of preparation because it implies the direct use of raw material. The plant cooked in several ways (16.76%) and condiments (16.25%) are the two categories that follow them.

As an example of alcoholic beverages, apart from punctual quotations, we collected 6 complete receipts of *ratafia* in the studied area, the most diverse in terms of plant taxa comprising 76 species. All of them (including the young *Juglans regia* fruits with a few incisions made) are put together in maceration in a big glass bottle with an anisate alcohol, conserved typically 40 days in an external part of the house, and then filtered and, if necessary, corrected in sugar. After this, and with a final graduation around 23°, it may be consumed as a pleasure and medicinal liqueur. Some of the plants ready to prepare *ratafia* are shown in Fig. 2.

To illustrate a few food elaborations, *Urtica dioica*, usually known as a medicinal plant—also employed as such in the studied area—appears as one of the wild plants with more variation: it may be scalded and then either seasoned and consumed as a vegetable or prepared in omelet, and it may be boiled and eaten in soup. *Sambucus nigra* flowers are consumed in a very common way in northeast Catalonian areas [10, 34], the so-called *brunyols* or *bunyols*. These are kind of pastry prepared coating the flowers with a pasta made with floor and water, frying them in very hot oil, and finally seasoning them with salt or sugar, depending on the use of the product with salty food or as a dessert. Additionally, and more originally, the flowers (optionally together with tender leaves) of this species are prepared and consumed in omelet.

For human food, the use of cultivated and wild plants is similar in percentages: 44.62% of the taxa used are cultivated and 55.38% are wild. Contrarily to what one could expect, with crops dominating the market, the relevance of wild food plants is high.

Concerning animal fodder (Table 5), the most reported families are Poaceae (153 UR), Fabaceae (105 UR), and Brassicaceae (70 UR) the five more cited species being *Zea mays* (56 UR), *Brassica napus* (46 UR), *Quercus ilex* (37 UR), *Medicago sativa* (34 UR), and *Triticum aestivum* (28 UR). In general, these species are consumed raw (43.88%) or air-dried and preserved (39.12%). The most used parts of plants are the aerial part that sometimes includes the whole plant (41.68%), leaves (19.74%), and seeds (14.1%). These grains can be given directly to the animals or processed in order to obtain flour or fodder.

**Table 5:** Fodder plants reported in the studied area

Family	Taxon (voucher)	Catalan vernacular names	Preparation	Used part	Wild (W)/cultivated (C)	Use reports
Amaranthaceae	*Beta vulgaris* L. subsp. *vulgaris* var. *crassa* (Alef.) Helm (BCN50761)	Bleda. Polpa (elaborated product). Remolatxa. Sucre (elaborated product). Sucre candi (elaborated product)	Air dried^a^. Boiled in water^a, e^. Raw^b, e^	Root. Whole plant	C	25
	*Beta vulgaris* L. subsp. *vulgaris* var. *vulgaris* (BCN46075)	Bleda	Boiled in water^e^	Aerial part	C	3
Apiaceae	*Foeniculum vulgare* Mill. (BCN26350)	Fonoll	Raw^f^	Aerial part	W	4
Aquifoliaceae	*Ilex aquifolium* L. (BCN29876)	Grèvol	Raw^f^	Leaf	W	1
Araceae	*Arum italicum* Mill. (BCN32358)	Xàrria. Xèrria	Boiled in water^e^. Raw	Root. Whole plant	W	4
Araliaceae	*Hedera helix* L. (BCN29869)	Heura. Heura d’alzina	Raw^a, g^	Leaf	W	3
Asparagaceae	*Agave americana* L. (BCN46860)	Figuerassa	Boiled in water^a, e^	Leaf	W	5
	*Aphyllanthes monspeliensis* L. (BCN29627)	Llonsa. Pa de cucut	Raw^a^	Aerial part	W	3
Asteraceae	*Centaurea jacea* L. (BCN21907)	Caps de burro	Raw^f^	Aerial part	W	1
	*Chondrilla juncea* L. (BCN29852)	Màstec	Raw^f^	Aerial part	W	2
	*Cichorium endivia* L. (BCN46854)	Escarola	Raw	Leaf	C	1
	*Cichorium intybus* L. (BCN29660)	Cama-roja. Xicoia. Xicoina. Xicòria	Raw^f^	Aerial part	W	2
	*Reichardia picroides* (L.) Roth (BCN113704)	Cosconilla	Raw^f^	Leaf	W	6
	*Silybum marianum* (L.) Gaertn. (BCN29958)	Card	Raw^b^	Aerial part	W	1
	*Sonchus oleraceus* L. (BCN113723)	Lletissó. Llipsó. Llistó	Air dried^f^. Raw^f, g^	Aerial part	W	5
	*Sonchus* sp.	Llepsó. Lletissó. Llistó	Raw^a, c, f^	Aerial part	W	5
	*Sonchus tenerrimus* L. (BCN29954)	Lletissó. Llitsó	Raw^f^	Aerial part	W	3
	*Taraxacum officinale* Weber in Wiggers (BCN25948)	Dent de lleó. Xicoia	Raw^f^	Aerial part	W	2
Brassicaceae	*Brassica napus* L. (BCN46856)	Nap. Nap de bou. Nap del camp	Boiled in water^a, e^. Raw^a, e^	Aerial part. Root. Whole plant	C	46
	*Brassica oleracea* L. subsp. *oleracea* (BCN32181)	Bròquil. Col. Col aloma	Boiled in water^a, e^. Raw^c, e, f^	Leaf	C	22
	*Capsella bursa-pastoris* (L.) Medic. (BCN46079)	Bossa de pastor. Caps blancs	Raw^f^	Aerial part	W	1
	*Raphanus raphanistrum* L. subsp. *sativus* (L.) Domin (BCN49860)	Rave	Raw^a^	Whole plant	C	1
Cannabaceae	*Celtis australis* L. (BCN29845)	Lledó (fruit). Lledoner	Boiled in water^e^. Raw^a, e^	Leaf	C	10
Caprifoliaceae	*Scabiosa atropurpurea* L. (BCN29947)	Escabiosa	Raw^f^	Aerial part	W	1
Convolvulaceae	*Convolvulus arvensis* L. (BCN29663)	Corretjola	Raw^f^	Aerial part	W	6
Cucurbitaceae	*Cucumis melo* L. (BCN46851)	Meló (fruit)	Raw^e^	Epicarp. Fruit	C	6
	*Cucurbita maxima* Duch. in Lam. (BCN-S-1499)	Rabequet (fruit). Carabassa (fruit)	Boiled in water^e^	Fruit	C	1
	*Cucurbita pepo* L. var. *oblonga* Link (BCN29859)	Carabassó (fruit)	Raw^e^	Fruit	C	1
	*Cucurbita pepo* L. var. *pepo* (BCN49858)	Carbassa (fruit). Carbassera. Rabequet (fruit)	Boiled in water^e^. Raw^e^	Fruit	C	12
Equisetaceae	*Equisetum arvense* L. (BCN24767)	Cua de cavall. Sangnua	Raw^f^	Aerial part	W	1
	*Equisetum* sp.	Cua de cavall. Sangnua	Raw^a, d^	Aerial part	W	3
Ericaceae	*Calluna vulgaris* (L.) Hull (BCN113722)	Bronsa. Bronsó	Raw^a^	Aerial part	W	1
Fabaceae	*Ceratonia siliqua* L. (BCN32177)	Garrofa (fruit)	Air-dried^a, d^	Fruit	C	8
	*Lupinus albus* L. (BCN64375)	Llobí	Raw^a, e^	Seed	C	3
	*Medicago sativa* L. (BCN29891)	Userda	Air-dried^a, b, d, e, f^. Boiled in water^a^. Raw^b, f^	Aerial part	C	34
	*Onobrychis viciifolia* Scop. (BCN113732)	Esparcet. Trepadella	Air-dried^a, e, f, g^. Raw^f^. Not reported^f^	Aerial part	C	20
	*Pisum sativum* L. (BCN32140)	Pèsol	Raw^c^	Fruit	C	1
	*Robinia pseudoacacia* L. (BCN31298)	Acàcia. Acàcia de jardí	Raw^b^	Leaf	C	1
	*Spartium junceum* L. (BCN29956)	Ginesta	Raw^b^	Aerial part	W	1
	*Trifolium incarnatum* L. (BCN25026)	Fenc	Air-dried^a, d^. Boiled in water^a^. Raw^a^	Aerial part	C	23
	*Trifolium pratense* L. (BCN29811)	Trèfola. Trèfoga	Air dried^a^. Raw^f^	Aerial part	W	4
	*Trigonella foenum-graecum* L. (BCN32120)	Senigrec	Raw	Aerial part	W	1
	*Vicia faba* L. (BCN46826)	Fava	Air-dried^d, e^. Raw^a^	Seed	C	6
	*Vicia sativa* L. (BCN47746)	Veça	Raw^a, f^	Aerial part	C	3
Fagaceae	*Castanea sativa* Mill. (BCN29844)	Castanya (fruit). Castanyer	Raw^e^	Fruit	C	1
	*Quercus ilex* L. (BCN113730)	Aglà (fruit). Alzina. Aulina. Gla (fruit)	Air dried^e^. Boiled in water^e^. Cooked^e^. Raw^a, e, f^	Fruit. Leaf. Young shoot	W	37
	*Quercus pubescens* Willd. (BCN30007)	Roure	Raw^a, e^	Aerial part	W	3
	*Quercus suber* L. (BCN46829)	Suro. Alzina surera	Raw^a^	Leaf	W	2
Linaceae	*Linum usitatissimum* L. (BCN47281)	Farina de llinet (elaborated product). Llinet	Air dried^e^	Seed	C	1
Malvaceae	*Malva sylvestris* L. (BCN29889)	Malva. Malva rosa	Raw^f^	Leaf	W	1
Moraceae	*Ficus carica* L. (BCN24887)	Figa (infructescence). Figa d’Alacant (infructescence). Figa de coll de senyora (infructescence). Figa de coll llarg blanca (infructescence). Figa de coll llarg negra (infructescence). Figa de pota de cavall (infructescence). Figa de Sant Joan (infructescence). Figa negra (infructescence). Figuera. Figuera de coll de senyora	Raw^e^	Infructescence	C	1
Oleaceae	*Fraxinus excelsior* L. (BCN46844)	Freixa	Raw^b, g^	Leaf	W	2
Passifloraceae	*Passiflora caerulea* L. (BCN29747)	Flor de crist	Raw^g^	Aerial part	C	1
Plantaginaceae	*Plantago lanceolata* L. (BCN32138)	Plantatge de fulla estreta. Plantatge estret	Raw^f^	Aerial part	W	1
	*Plantago major* L. (BCN29910)	Plantatge. Plantatge ample. Plantatge de fulla ampla	Raw^f^	Aerial part. Leaf. Whole plant	W	6
	*Plantago* sp.	Plantatge	Raw^f^	Leaf	W	2
Poaceae	*Arundo donax* L. (BCN29825)	Canya. Canya americana. Canyer	Raw^a, b^	Leaf	W	2
	*Avena sativa* L. (BCN29839)	Civada	Air dried^a, c, f^. Raw^b^	Aerial part. Fruit	C	26
	*Cynodon dactylon* (L.) Pers (BCN29686)	Gram	Raw^a^	Aerial part	W	1
	*Digitaria sanguinalis* (L.) Scop. (BCN113745)	Forcadella. Xereix	Raw^f^	Aerial part	W	2
	*Hordeum vulgare* L. (BCN46843)	Ordi	Air dried^c, g^. Boiled in water^e^	Aerial part. Fruit	C	10
	*Lolium perenne* L. (BCN58204)	Margall. Raigràs	Air dried^a, g^. Raw^b, f^	Aerial part	C	10
	*Panicum miliaceum* L. (BCN12911)	Mill	Air dried^c^	Fruit	C	5
	*Phalaris arundinacea* L. (BCN51675)	–	Raw^b^	Aerial part	C	1
	*Secale cereale* L. (BCN46828)	Sègol. Sègal	Air dried^a^. Boiled in water^a^. Raw^b^	Aerial part. Fruit	C	6
	*Sorghum bicolor* (L.) Moench (BCN31310)	Melca. Sorgo	Air dried^a, c, d^. Raw^a^	Aerial part	C	6
	*Triticum aestivum* L. (BCN29963)	Blat. Farina (elaborated product). Pa (elaborated product). Palla (elaborated product). Segó (bran)	Air dried^a, b, c, d, e^. Boiled in water^c, e^. Cooked^f^	Aerial part. Bran. Fruit	C	28
	*Zea mays* L. (BCN29830)	Blat de morassa. Blat de moret. Blat de moro. Farro (elaborated product)	Air dried^a, c, d, e, f, g^. Boiled in water^c, e^. Raw^a^	Aerial part. Bract. Fruit	C	56
Polygonaceae	*Fagopyrum esculentum* Moench (BCN24886)	Fajol	Boiled in water	Seed	C	1
Portulacaceae	*Portulaca oleracea* L. (BCN46835)	Verdolaga	Raw^a, e, f^	Aerial part	W	11
Primulaceae	*Anagallis arvensis* L. (BCN29974)	Marruc	Raw^c^	Aerial part	W	2
Ranunculaceae	*Anemone hepatica* L. (BCN29834)	Herba fetgera	Raw^c^	Leaf	W	2
	*Clematis flammula* L. (BCN29856)	Viadella. Virobella	Air dried^a^	Leaf	W	3
	*Clematis recta* L. (BCN113720)	Viadella	Air dried^a^	Aerial part	W	1
	*Clematis vitalba* L. (BCN29857)	Ridorta	Air dried^a^. Raw^g^	Leaf	W	2
Rosaceae	*Rubus ulmifolius* Schott (BCN29938)	Bardissa. Mora (fruit). Mora negra (fruit). Romeguera	Raw^b^	Young shoot	W	1
Smilacaceae	*Sanguisorba minor* Scop. (BCN113728)	Esparcet bord	Raw^a^	Aerial part	W	1
	*Smilax aspera* L. (BCN29951)	Arítjol	Raw^f^	Aerial part	W	3
Solanaceae	*Solanum lycopersicum* L. (BCN29952)	Tomata (fruit). Tomata de guardar (fruit). Tomata de la meta (fruit). Tomata de penjar (fruit). Tomata dels tres *cantos* (fruit). Tomata plena (fruit). Tomata poma (fruit)	Raw^c^	Fruit	C	1
	*Solanum tuberosum* L. (BCN29797)	Patata. Patatera. Trumfera	Boiled in water^c, e^. Raw	Tuber	C	5
Ulmaceae	*Ulmus minor* Mill. (BCN113729)	Om	Boiled in water^e^. Raw^a, b, e, f^	Leaf	W	10

Fodder destination: ^a^Cows, ^b^Goats, ^c^Hens, ^d^Horses and mares, ^e^Pigs, ^f^Rabbits, ^g^Sheep; without superscripted letter: animal destination is not clear

For animal feed, the percentage repartition is similar than for human food: 44.44% of taxa used are cultivated and 55.56% are wild, again accounting for the importance of food plants in the region considered.

### Other uses

This category, arranged in Table 6, includes uses that are neither medicinal or food. This is a melting pot with numerous subcategories. Most probably, in societies currently much more dependent on natural resources at an ethnobotanical level, many subcategories could be treated independently, because they would receive a big number of use reports, but we have realized that in our cultural area, where many uses have only few reports based on ancient memories, it is practical to treat all of them together (Gras et al. 2016). We have collected 894 UR concerning 125 taxa, 8 of them only determined at the genus level. These taxa belong to 47 plant families, Fagaceae (136 UR, 15.21%), Poaceae (117 UR; 13.09%), Ericaceae (109 UR; 12.19%), Cannabaceae (69 UR; 7.72%), and Fabaceae (48 UR; 5.37%) being the most cited.

**Table 6:** Plant with other uses reported in the studied area

Family	Taxon (voucher)	Catalan vernacular names	Use	Used part	Use reports
Adoxaceae	*Sambucus nigra* L. (BCN113595)	Sabuquer. Saüc. Saüquer	Artisanal. Fuel obtaining	Stem	14
	*Viburnum tinus* L. (BCN30012)	Marfull	Ornamental	Whole plant	1
Apiaceae	*Pimpinella anisum* L. (BCN47278)	Anís verd. Matafaluga	Repellent	Whole plant	1
Araceae	*Arum italicum* Mill. (BCN32358)	Xàrria. Xèrria	Agrosilvopastoral management	Flower	1
Araliaceae	*Hedera helix* L. (BCN29869)	Heura. Heura d’alzina	Ornamental	Whole plant	1
Arecaceae	*Phoenix dactylifera* L. (BCN52783)	Palma	Magic and religious beliefs and practices	Leaf	5
Asparagaceae	*Agave americana* L. (BCN46860)	Figuerassa	Unclassified	Inflorescence	1
	*Asparagus acutifolius* L. (BCN29976)	Espàrgol. Esparreguera. Espàrrec	Folk oral literature. Ornamental	Aerial part. Young shoot	2
	*Ruscus aculeatus* L. (BCN29939)	Galzeran. Galleranc	Ornamental	Aerial part	3
	*Yucca aloifolia* L. (BCN286)	–	Ornamental. Unclassified	Whole plant	2
Asteraceae	*Carlina acanthifolia* All. (BCN24738)	Cardina. Carlina. Carolina	Domestic. Ornamental	Whole plant	8
	*Helichrysum stoechas* (L.) Moench (BCN29872)	Mançanilla. Sempreviva	Ornamental	Aerial part	2
	*Mantisalca salmantica* (L.) Briq. et Cavill. (BCN24925)	Baleja	Artisanal	Aerial part	1
	*Santolina chamaecyparissus* L. (BCN113709)	Espernallac. Santolina	Ornamental	Whole plant	1
	*Sonchus* sp.	Llepsó. Lletissó. Llistó	Agrosilvopastoral management. Unclassified	Aerial part. Whole plant	2
	*Taraxacum officinale* Weber in Wiggers (BCN25948)	Dent de lleó. Xicoia	Ludic	Infructescence	2
Begoniaceae	*Begonia* sp.	Tamaia	Ornamental	Whole plant	1
Betulaceae	*Alnus glutinosa* (L.) Gaertn. (BCN29620)	Vern	Artisanal. Timber	Stem	4
	*Corylus avellana* L. (BCN29831)	Avellana (fruit). Avellana del queixal (fruit). Avellaner. Avellaner negret	Agrosilvopastoral management. Artisanal. Fuel obtaining. Timber	Stem	15
Boraginaceae	*Lithospermum officinale* L. (BCN113576)	Herba pedrera	Ornamental	Aerial part	1
Buxaceae	*Buxus sempervirens* L. (BCN29843)	Boix	Agrosilvopastoral management. Artisanal. Timber	Aerial part. Stem	14
Cannabaceae	*Cannabis sativa* L. (BCN24735)	Cànem. Carm	Artisanal. Textile	Stem	6
	*Celtis australis* L. (BCN29845)	Lledó (fruit). Lledoner	Agrosilvopastoral management. Artisanal. Timber	Fruit. Stem	63
Cistaceae	*Cistus albidus* L. (BCN36672)	Estepa	Smoking plant	Leaf	1
	*Cistus monspeliensis* L. (BCN36740)	Estepa. Mòdega	Artisanal	Aerial part	2
	*Cistus salviifolius* L. (BCN36767)	Estepa. Mòdega	Artisanal. Domestic	Aerial part	4
Coriariaceae	*Coriaria myrtifolia* L. (BCN113731)	Roldor	Agrosilvopastoral management. Artisanal. Magic and religious beliefs and practices	Aerial part. Stem	7
Cucurbitaceae	*Cucurbita pepo* L. var. *pepo* (BCN49858)	Carbassa (fruit). Carbassera. Rabequet (fruit)	Artisanal	Fruit	1
Cupressaceae	*Cupressus sempervirens* L. (BCN35770)	Xiprer	Folk oral literature	Whole plant	2
	*Juniperus communis* L. (BCN113589)	Ginebre. Ginebró	Timber	Stem	1
	*Juniperus oxycedrus* L. (BCN29879)	Càdec	Artisanal. Domestic. Timber	Fruit. Stem	4
Dennstaedtiaceae	*Pteridium aquilinum* (L.) Kuhn (BCN113735)	Falguera	Agrosilvopastoral management	Frond	5
Dryopteridaceae	*Dryopteris filix-mas* (L.) Schott (BCN29629)	Falguera	Agrosilvopastoral management	Frond	8
Equisetaceae	*Equisetum arvense* L. (BCN24767)	Cua de cavall. Sangnua	Agrosilvopastoral management	Aerial part	1
	*Equisetum* sp.	Cua de cavall. Sangnua	Agrosilvopastoral management	Aerial part	1
Ericaceae	*Arbutus unedo* L. (BCN29836)	Arboç. Bola d’arboç (fruit). Cirera d’arboç (fruit). Cirerer d’arboç	Agrosilvopastoral management. Artisanal. Fuel obtaining. Not reported	Stem	11
	*Calluna vulgaris* (L.) Hull (BCN113722)	Bronsa. Bronsó	Agrosilvopastoral management. Fuel obtaining	Aerial part	4
	*Erica arborea* L. (BCN113593)	Bruc. Bruc boal. Bruc bord. Bruc d’ull de bou. Bruc de bou. Bruc de llei	Agrosilvopastoral management. Artisanal. Fuel obtaining. Timber. Unclassified	Aerial part. Root. Stem	38
	*Erica multiflora* L. (BCN29864)	Bruc	Agrosilvopastoral management. Artisanal. Fuel obtaining. Unclassified	Aerial part	4
	*Erica scoparia* L. (BCN113724)	Bruc. Bruc bord. Bruc d’escombres. Bruc de llei	Agrosilvopastoral management. Artisanal. Domestic. Fuel obtaining. Unclassified	Aerial part. Stem. Whole plant	44
	*Erica* sp.	Bruc	Agrosilvopastoral management. Artisanal. Fuel obtaining.	Aerial part. Stem	8
Fabaceae	*Genista scorpius* (L.) DC. in Lam. et DC. (BCN27292)	Argelaga. Espines	Agrosilvopastoral management. Fuel obtaining.	Aerial part	13
	*Medicago sativa* L. (BCN29891)	Userda	Agrosilvopastoral management	Aerial part	1
	*Onobrychis viciifolia* Scop. (BCN113732)	Esparcet. Trepadella	Agrosilvopastoral management	Flower	1
	*Phaseolus vulgaris* L. (BCN46837)	Fesol de l’ull *marrón*. Fesol de l’ull negre. Fesol de Santa Pau. Fesol del bitxet gros. Fesol de bitxet petit. Fesol menut. Fesola. Mongeta	Agrosilvopastoral management	Whole plant	1
	*Robinia pseudoacacia* L. (BCN31298)	Acàcia. Acàcia de jardí	Agrosilvopastoral management. Ornamental. Timber	Stem. Whole plant	13
	*Spartium junceum* L. (BCN29956)	Ginesta	Agrosilvopastoral management. Fuel obtaining. Magic and religious beliefs and practices. Timber	Aerial part. Flower. Stem	12
	*Trifolium incarnatum* L. (BCN25026)	Fenc	Agrosilvopastoral management	Aerial part. Flower	2
	*Trigonella foenum-graecum* L. (BCN32120)	Senigrec	Repellent	Whole plant	1
	*Ulex parviflorus* Pourr. (BCN30011)	Gatosa	Agrosilvopastoral management. Fuel obtaining	Aerial part	3
	*Wisteria sinensis* (Sims) Sweet (BCN30014)	Lilà	Ornamental	Whole plant	1
	*Castanea sativa* Mill. (BCN29844)	Castanya (fruit). Castanyer	Agrosilvopastoral management. Artisanal. Timber	Stem	28
Fagaceae	*Fagus sylvatica* L. (BCN46845)	Faig	Fuel obtaining	Stem	1
	*Quercus coccifera* L. (BCN29765)	Garrigues	Domestic	Aerial part	1
	*Quercus ilex* L. (BCN113730)	Aglà (fruit). Alzina. Aulina. Gla (fruit)	Agrosilvopastoral management. Artisanal. Dyer. Fuel obtaining. Ludic. Magic and religious beliefs and practices. Not reported. Tannery. Timber. Unclassified	Aerial part. Bark. Flower. Fruit. Leaf. Stem	66
	*Quercus pubescens* Willd. (BCN30007)	Roure	Agrosilvopastoral management. Folk oral literature. Fuel obtaining. Timber	Fruit. Leaf. Stem	25
	*Quercus suber* L. (BCN46829)	Suro. Alzina surera	Agrosilvopastoral management. Domestic. Fuel obtaining. Ludic. Textile. Timber. Unclassified.	Bark. Stem. Whole plant	15
Geraniaceae	*Pelargonium* sp.	Gerani	Ornamental	Whole plant	1
Juglandaceae	*Juglans regia* L. (BCN29877)	Noguer. Nou (fruit). Nou verda (fruit)	Dyer. Folk oral literature. Magic and religious beliefs and practices	Fruit. Whole plant	5
Juncaceae	*Juncus effusus* L. (BCN39991)	Jonc	Agrosilvopastoral management	Stem	1
Lamiaceae	*Lavandula latifolia* Medic. (BCN113740)	Barballó. Espígol. Lavanda	Cosmetic. Domestic. Ornamental	Aerial part. Whole plant	5
	*Ocimum basilicum* L. (BCN29897)	Alfàbrega	Agrosilvopastoral management. Repellent	Whole plant	4
	*Origanum vulgare* L. (BCN113705)	Orenga	Folk oral literature	Whole plant	2
	*Rosmarinus officinalis* L. (BCN113599)	Romaní	Domestic. Folk oral literature. Magic and religious beliefs and practices	Aerial part	14
	*Salvia farinacea* Benth. (BCN113718)	Sàlvia de jardí	Ornamental	Whole plant	1
	*Salvia officinalis* L. subsp. *officinalis* (BCN113583)	Sàlvia. Sàlvia de fulla ampla	Ornamental	Whole plant	1
	*Thymus* ×*citriodorus* (Pers.) Schreber (BCN113803)	Farigola de xocolata. Farigola llimonera	Ornamental	Whole plant	1
	*Thymus vulgaris* L. (BCN113590)	Farigola	Folk oral literature. Magic and religious beliefs and practices	Aerial part. Whole plant	4
Lauraceae	*Laurus nobilis* L. (BCN113717)	Llord. Llorer	Magic and religious beliefs and practices	Aerial part	39
Lythraceae	*Punica granatum* L. (BCN29764)	Magrana (fruit). Magraner. Magraner agre. Magraner bord. Magraner dolç	Artisanal. Magic and religious beliefs and practices	Aerial part	21
Moraceae	*Ficus carica* L. (BCN24887)	Figa (infructescence). Figa d’Alacant (infructescence). Figa de coll de senyora (infructescence). Figa de coll llarg blanca (infructescence). Figa de coll llarg negra (infructescence). Figa de pota de cavall (infructescence). Figa de Sant Joan (infructescence). Figa negra (infructescence). Figuera. Figuera de coll de senyora	Magic and religious beliefs and practices. Folk oral literature	Infructescence. Whole plant	7
	*Morus alba* L. (BCN52588)	Morera	Agrosilvopastoral management	Stem	2
	*Morus nigra* L. (BCN31289)	Arça. Morera	Agrosilvopastoral management. Fuel obtaining	Stem	3
Myrtaceae	*Eucalyptus globulus* Labill. (BCN29696)	Eucaliptu. Eucaliptus	Ornamental	Aerial part	1
Oleaceae	*Fraxinus excelsior* L. (BCN46844)	Freixa	Timber	Stem	3
	*Olea europaea* L. subsp. *europaea* (BCN29898)	Oli (elaborated product). Oli d’oliva (elaborated product). Olivera. Oliva (fruit)	Artisanal. Domestic. Folk oral literature. Fuel obtaining. Magic and religious beliefs and practices. Timber	Aerial part. Fruit. Stem	10
Papaveraceae	*Papaver rhoeas* L. (BCN29903)	Gallaret. Pipiripip. Quiquiriquí. Rosella	Ludic. Magic and religious beliefs and practices	Flower	13
Pinaceae	*Pinus halepensis* Mill. (BCN113592)	Pi. Pi blanc. Pi bord. Pi de pinya llarga. Pi petit. Pinya (fructification)	Agrosilvopastoral management. Artisanal. Folk oral literature. Fuel obtaining. Timber	Aerial part. Bark. Fructification. Stem. Whole plant	20
	*Pinus pinaster* Ait. (BCN36559)	Pi bord. Pi melis	Agrosilvopastoral management. Fuel obtaining	Stem	4
	*Pinus pinea* L. (BCN26751)	Pi. Pi de llei. Pi de pinya. Pi pinyer	Fuel obtaining	Fructification	1
	*Pinus* sp.	Pi. Trementina (elaborated product)	Agrosilvopastoral management. Artisanal. Domestic. Fuel obtaining. Timber	Aerial part. Cortical parenchyma. Leaf. Stem	9
Poaceae	*Arundo donax* L. (BCN29825)	Canya. Canya americana. Canyer	Agrosilvopastoral management. Artisanal	Leaf. Stem. Whole plant	48
	*Avena barbata* Pott ex Link in Schrad. (BCN49867)	Avena. Cugula	Ludic	Fruit	16
	*Avena sativa* L. (BCN29839)	Civada	Agrosilvopastoral management. Ludic	Aerial part. Fruit	5
	*Briza media* L. (BCN113733)	Belluguets	Ornamental	Flower	2
	*Panicum miliaceum* L. (BCN12911)	Mill	Magic and religious beliefs and practices	Fruit	1
	*Phragmites australis* (Cav.) Steudel (BCN27104)	–	Artisanal	Stem	1
	*Secale cereale* L. (BCN46828)	Sègol. Sègal	Agrosilvopastoral management	Aerial part	2
	*Sorghum bicolor* (L.) Moench (BCN31310)	Melca. Sorgo	Artisanal	Aerial part	2
	*Stipa tenacissima* L. (BCN46091)	Espart	Textile	Aerial part. Stem	3
	*Triticum aestivum* L. (BCN29963)	Blat. Farina (elaborated product). Pa (elaborated product). Palla (elaborated product). Segó (bran)	Agrosilvopastoral management	Fruit	6
	*Zea mays* L. (BCN29830)	Blat de morassa. Blat de moret. Blat de moro. Farro (elaborated product)	Agrosilvopastoral management. Artisanal. Domestic. Folk oral literature. Fuel obtaining. Ludic. Ornamental. Textile	Bract. Fruit. Inflorescence.Stem. Styles and stigmas	31
Ranunculaceae	*Clematis flammula* L. (BCN29856)	Viadella. Virobella	Agrosilvopastoral management	Aerial part	2
	*Clematis vitalba* L. (BCN29857)	Ridorta	Agrosilvopastoral management. Domestic. Textile	Aerial part. Stem	6
Rosaceae	*Crataegus monogyna* Jacq. (BCN29858)	Arç. Arç blanc	Agrosilvopastoral management. Fuel obtaining	Stem. Whole plant	5
	*Cydonia oblonga* Mill. (BCN46849)	Codony (fruit). Codonyat (elaborated product). Codonyer	Agrosilvopastoral management	Whole plant	3
	*Mespilus germanica* L. (BCN50768)	Nespler. Nespla de bosc. Nespra. Nespro	Agrosilvopastoral management	Whole plant	1
	*Prunus armeniaca* L. (BCN48712)	Abricoc (fruit). Albercoc (fruit). Albercoquer	Agrosilvopastoral management. Artisanal	Endocarp. Whole plant	3
	*Prunus avium* (L.) L. (BCN29827)	Cirera (fruit). Cirerer	Agrosilvopastoral management	Stem. Whole plant	2
	*Prunus dulcis* (Mill.) Weeb. (BCN46833)	Ametller	Agrosilvopastoral management	Whole plant	1
	*Prunus persica* (L.) Batsch (BCN46832)	Préssec (fruit). Préssec cardinal (fruit). Préssec de coure (fruit). Préssec duran (fruit). Préssec groc (fruit). Préssec groc d’agost (fruit). Préssec mollar (fruit). Préssec sang de llebre (fruit). Préssec de Sant Joan (fruit). Préssec de Sant Pere (fruit). Presseguer	Agrosilvopastoral management	Whole plant	3
	*Prunus spinosa* L. (BCN30005)	Aranyó (fruit). Aranyoner. Arç. Arç negre. Arça	Domestic	Whole plant	1
	*Pyrus communis* L. subsp. *communis* (BCN46831)	Pera (fruit). Pera *conference* (fruit). Pera de Sant Joan (fruit). Pera rogija (fruit). Perer. Perer mau	Agrosilvopastoral management	Whole plant	2
	*Pyrus malus* L. subsp. *mitis* (Wallr.) O.Bolòs et J.Vigo (BCN46830)	Poma (fruit). Poma aspra (fruit). Poma cambusina (fruit). Poma camosa (fruit). Poma capçana (fruit). Poma del ciri (fruit). Poma del ciri groga (fruit). Poma del ciri vermella (fruit). Poma *golden* (fruit). Poma rodona (fruit). Poma *royal* (fruit). Pomer. Pomer del ciri. Pomera. Pomera del ciri	Agrosilvopastoral management. Domestic	Fruit. Whole plant	11
	*Rosa* sp.	Rosa. Rosa de jardí. Roser	Ornamental	Whole plant	1
	*Rubus ulmifolius* Schott (BCN29938)	Bardissa. Mora (fruit). Mora negra (fruit). Romeguera	Folk oral literature. Fuel obtaining	Aerial part. Young shoot	2
Rutaceae	*Citrus aurantium* L. (BCN46080)	Taronger agre. Taronger amarg. Taronger bord. Taronja agra (fruit)	Agrosilvopastoral management	Fruit. Whole plant	3
	*Citrus japonica* Thunb. (BCN113966)	Llimona de Xipre	Ornamental	Whole plant	1
	*Citrus limon* (L.) Burm. (BCN46853)	Llimona (fruit). Llimoner	Agrosilvopastoral management	Fruit	1
	*Citrus sinensis* (L.) Osbeck (BCN24752)	Taronger. Taronger dolç. Taronja (fruit)	Agrosilvopastoral management	Whole plant	1
	*Ruta chalepensis* L. (BCN29940)	Ruda	Domestic. Folk oral literature. Magic and religious beliefs and practices	Aerial part. Whole plant	3
Salicaceae	*Populus* ×*canadensis* Moench (BCN113967)	Arbre. Pollancre	Timber	Stem	1
	*Populus nigra* L. (BCN113746)	Arbre. Arbre bord. Pollancre	Agrosilvopastoral management. Artisanal. Timber	Stem	3
	*Salix alba* L. (BCN29777)	Sàlix. Saule	Artisanal	Stem	7
	*Salix fragilis* L. (BCN31305)	Vimbera. Vímec. Vimequera. Vim	Artisanal	Stem	17
Sapindaceae	*Aesculus hippocastanum* L. (BCN29618)	Castanyer bord	Timber	Stem	1
Saxifragaceae	*Bergenia* sp.	Hortènsia d’hivern	Ornamental	Whole plant	1
Smilacaceae	*Smilax aspera* L. (BCN29951)	Arítjol	Fuel obtaining	Aerial part	1
Solanaceae	*Nicotiana tabacum* L. (BCN48711)	Tabac	Agrosilvopastoral management. Repellent. Smoking plant	Leaf	3
	*Solanum tuberosum* L. (BCN29797)	Patata. Patatera. Trumfera	Agrosilvopastoral management. Domestic. Smoking plant	Leaf. Tuber. Whole plant	4
Typhaceae	*Typha latifolia* L. (BCN31314)	Balca	Agrosilvopastoral management. Artisanal	Stem	10
Ulmaceae	*Ulmus minor* Mill. (BCN113729)	Om	Agrosilvopastoral management. Artisanal. Timber	Stem	13
Urticaceae	*Urtica dioica* L. (BCN29814)	Ortiga	Agrosilvopastoral management. Folk oral literature. Not reported	Aerial part. Whole plant	9
Violaceae	*Viola alba* Besser (BCN27286)	Viola. Violeta	Ornamental	Aerial part	1
Vitaceae	*Vitis vinifera* L. (BCN29972)	Raïm (fruit). Sarment. Vi (elaborated product). Vinagre (elaborated product). Vinya	Not reported	Fruit	1

The five most reported species are *Quercus ilex* (66 UR; 7.38%), *Celtis australis* (63 UR; 7.05%), *Arundo donax* (48 UR; 5.37%), *Erica scoparia* (44 UR; 4.92%), and *Laurus nobilis* (39 UR; 4.36%). This top list reflects the persistency of a rural bottom still alive in the studied territory, since these plants are importantly used for agricultural practices, e.g., *Arundo* to grow *Lycopersicon esculentum* or *Phaseolus vulgaris* plants, *Celtis* to elaborate forks and *Erica* (as its specific epithet claims, indicating an old use) to make brooms (Fig. 2).

**Fig. 4:**
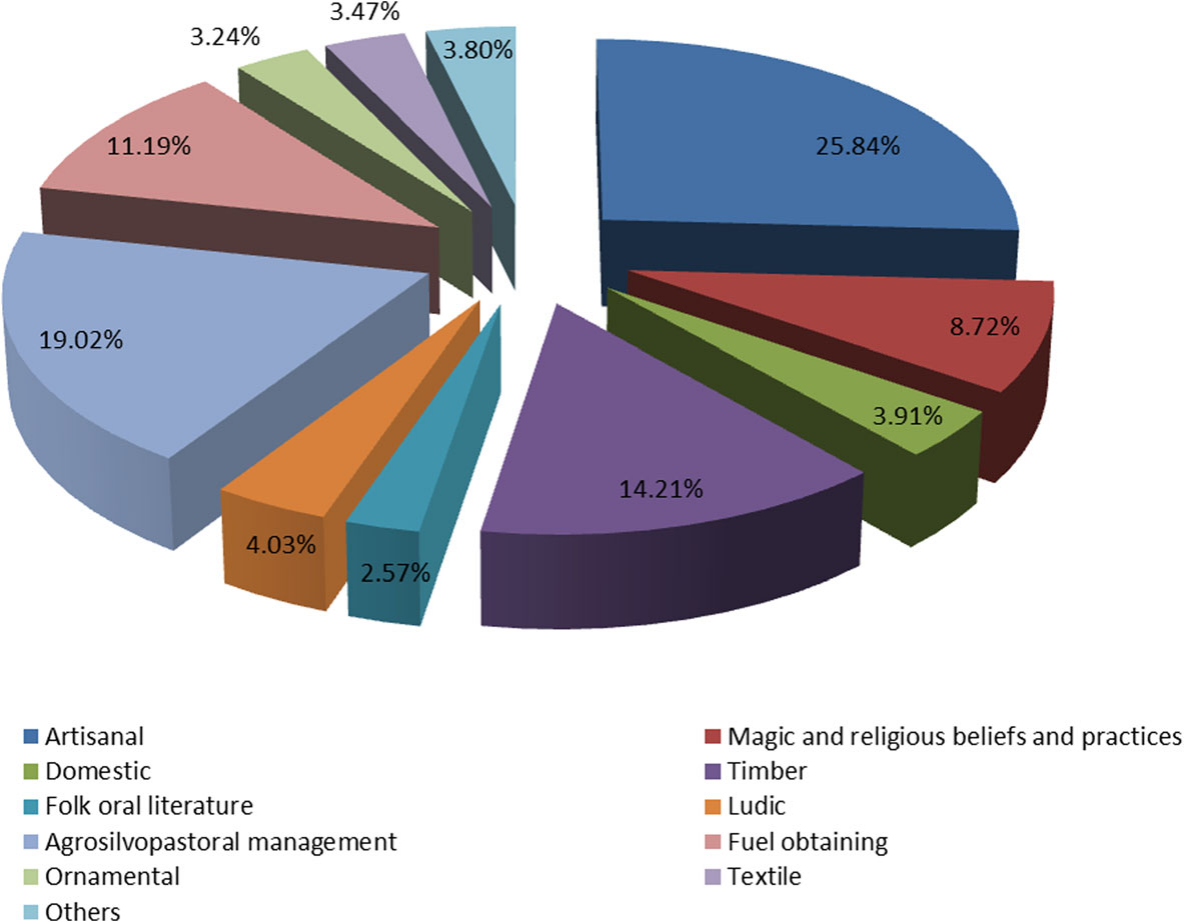
Other uses, as a percentage, quoted in Gironès

Even if we treat all of them in a single category, thus comparable with the medicinal and food ones, the different uses (subcategories) are also addressed, and those regarding the present study can be observed in Fig. 4. We emphasize the importance of the artisanal uses (231 UR, 25.84%) comprising the making of shoes, toys, and brooms among others, agrosilvopastoral management (170 UR, 19.02%) and timber (127 UR, 14.21%). Some of these categories correspond to professions that no longer exist but that have had a lot of relevance in the past and are, in some cases, transformed to sell their products as touristic objects.

Regarding the parts of plant used, which are closely related with their uses, the most reported are the stem and trunk (364 UR; 40.72%), followed by the aerial part and the whole plant (253 UR; 28.3%), and the fruit and infructescence (65 UR; 8.50%).

### Vernacular names

In this study, 581 vernacular names for 306 species, subspecies, and varieties have been collected, comprising 2892 reports. Ten taxa have been mentioned by the informants without any popular name. This is a very small percentage of the phytonyms recorded, and reflects that, in a few cases, the informants do not know (or, more often, do not remember) the name of a plant. The most reported taxa are those with a single or a few vernacular names as a general trend, as is the case of thyme (*Thymus vulgaris*), which has been mentioned 52 times with a unique popular name (*farigola* in Catalan language). In contrast, the species designated with more vernacular names are the cultivated ones, due to the names of landraces of several taxa that have also been quoted by the informants.

The ethnophytonymy index [53] shows a high value (32.6%), meaning that roughly one-third of the plants in the studied area have at least one folk name, comparable to the results from other Catalan territories, such as 35% in the Pallars district [5], 31% in Alt Empordà district [10], 29.8% in the island of Mallorca [12], 28% in the Montseny mountain massif [6], and 18% in Castelló province [1]. The allochthonous ethophytonymy index proposed by Carrió [12] calculates the rate between taxa having a vernacular name in non-Catalan languages (even for those taxa having also some Catalan names) and the total number of collected taxa. In the present study, the value is very small (4.7%, due to a few Spanish names) as compared with the one obtained in Mallorca (27.8, due to some names in Spanish and French languages; [12]), indicating a more culturally homogeneous informants’ pool.

The linguistic diversity index, which expresses the linguistic richness of a territory independently of its flora, reaches a value of 1.90 (almost two names per plant, in mean), comparable to those obtained in l’Alt Empordà (1.94) [10], Navarra (1.87) [75], and Montseny (1.76) [6].

It is interesting to remark that a certain number of folk plant names are linked to their uses. For instance, *Achillea ageratum* is called in Catalan language *herba del fàstic* (“disgusting herb”), since it incites vomiting; *herba cuquera* (“worm’s herb”) and *herba fetgera* (“liver’s herb”) allude, respectively, to the antihelminthic use of *Agrimonia eupatoria* and the hepatoprotective use of *Anemone hepatica* (the latter bearing the same indication in its specific epithet); *Centaurea aspera*, an hypoglycemiant plant, is named *flor del sucre* (“sugar’s flower”); *nap de bou* (“cow’s rape”) announces the use of *Brassica napus* to feed cattle; *bruc d’escombres* (“broom’s heather”) confirms the specific epithet of *Erica scoparia*, which is used, as other *Erica* species, for broom elaboration. All kind of plant uses are reflected in some vernacular names. We believe that an in-depth research on folk phytonyms (in different areas of a language and in different languages) and of scientific plant names that reflect plant uses is an interesting field of research, still scarcely or not at all addressed in ethnobotany.

## Concluding remarks

This study has revealed that traditional knowledge is persisting in the studied area if we take into account the numbers of taxa quoted and of use reports, as well as the values of the calculated indexes and despite the proximity to the highly urbanized areas. We have detected a significant number of allochthonous useful plants, and we believe that this subject should be particularly addressed in ethnobotanical studies in other areas throughout the world. The food plant use dataset is particularly important. Conversely, although the knowledge remains in the memory of our informants, the medicinal use of plants is substantially smaller than it used to be (informants often speak in the past of these uses) in their daily life, proving the erosive process in plant traditional knowledge and use that our industrialized societies are experiencing. In this sense, our research helps to alleviate this deterioration and to inventory this heritage, making ready for dissemination and reintroduction to younger generations of the society, who have suffered acculturation, and also for further studies in drug or other useful products development. In any case, even though the current ethnopharmacological pool is eroded and less employed as opposed to some decades ago, as we have shown, the number of medicinal plants and uses recorded are clearly higher than in less industrialized areas, where uses are more persistent. This applies, even more, for the food ethnobotanical corpus and, again to a lesser extent, to the ethnobotany of non-food and non-medicinal plant uses, finally showing the general solidity of ethnobotanical tradition in the area studied, which is now recorded and, thus, protected.
